# Histone methyltransferase G9a crosstalks with H3K36 histone methyltransferases NSD3 and SETD2 to mediate gene activation

**DOI:** 10.3389/fcell.2026.1790894

**Published:** 2026-03-26

**Authors:** Arunim Shah, Chandra Prakash Chaturvedi, Kulwant Singh, Shobhita Katiyar, Akhilesh Sharma

**Affiliations:** Stem Cell Research Center, Department of Hematology, Sanjay Gandhi Postgraduate Institute of Medical Sciences (SGPGIMS), Lucknow, India

**Keywords:** coactivator, G9a, NSD3, SETD2, *β-globin* locus

## Abstract

The euchromatic histone methyltransferase G9a/KMT1C/EHMT-II functions as a corepressor and a coactivator of transcription, depending upon its association with distinct protein complexes. While the mechanisms of G9a-mediated repression are well understood, the molecular mechanism of G9a-mediated activation remains elusive. In the present study, we report that the coactivator function of G9a involves its association with H3K36 histone methyltransferases, including NSD3/KMT3F/WHSC1L1 and SETD2/KMT3A. Functionally, we demonstrate that the association of G9a with NSD3 and SETD2 is necessary for activating G9a target adult *β* ^*major*^ *globin* and *β* ^*minor*^ *globin* genes in differentiating adult erythroid cells. Mechanistically, G9a recruits NSD3 to the *β* ^*major*^ *globin* gene, and NSD3 activates its expression by stabilizing Mediator complex binding to the promoter and facilitating SETD2-mediated H3K36 trimethylation in the gene body. Knocking down either NSD3 or G9a in differentiating erythroid cells significantly downregulates mediator complex binding at the promoter and the localization of SETD2 and SETD2-mediated H3K36me3 on the coding region of the G9a target *β* ^*major*^ *globin gene,* highlighting the necessity of NSD3 in mediating the activation of this gene. Our study reveals a novel crosstalk mechanism in which the histone methyltransferase G9a coordinates with the H3K36 histone methyltransferases NSD3 and SETD2 to mediate gene activation.

## Introduction

Histones’ post-translational modifications (PTMs) are essential in regulating gene expression during mammalian development (Bannister and Kouzarides, 2011; Rinaldi and Benitah, 2015). Various PTMs have been reported to date; among these, histone lysine methylation is the most common PTM, carried out by specific enzymes called histone methyltransferases (HMTs), while histone demethylases (HDMs) act in opposition to erase these methyl marks. Histone lysine methylation can be categorized into various histone lysine methylation sites, including H3K4, H3K9, H3K27, H3K36, and H3K79 (Dambacher et al., 2010; Hyun et al., 2017; Morishita et al., 2014). Each of these histone lysine modifications has a different effect on chromatin structure and transcriptional outcome. Likewise, the methylations of H3K4, H3K36, and H3K79 are indicative of active chromatin regions, whereas methylation of H3K9 and H3K27 are marks of repressive chromatin (Hyun et al., 2017). Various HMTs and HDMs that regulate these modifications have been reported. For example, H3K9 and H3K36 methylation, are mediated by H3K9 methyltransferases (Suv39H/KMT1A-B, G9a/KMT1C, and GLP/KMT1D) and H3K36 methyltransferases (SETD2/KMT3A, NSD1/KMT2B, NSD2/KMT3G, NSD3/KMT3F), respectively. Similarly, the HDMs that erase these marks include H3K9 demethylases (JHDM2A-C/KDM3A-C, JHDM3B/KDM4B, JHDM3C/KDM4C), and H3K36 demethylases (JHDM1A-1B/KDM2A-2B, JHDM3A-3C/KDM4A-4C) respectively (Hyun et al., 2017; Morishita et al., 2014). While these HMTs and HDMs can individually regulate gene activation or repression, growing evidence suggests that coordination and crosstalk among them are also vital for gene expression fate (Hosey et al., 2010; Suganuma and Workman, 2008). The crosstalk between these modifications can influence one another, such that one modification can recruit or activate chromatin-modifying complexes to generate a different histone modification (Zhang et al., 2015; Li and Wang, 2017). Many vital crosstalks between histone-modifying enzymes, which play a regulatory role in mediating gene activation and repression, have been described (Zhang et al., 2015; Chaturvedi et al., 2012). For example, the crosstalk between histone H3K4 methyltransferases MLL3/MLL4 and H3K27 demethylase UTX mediates gene activation (Suganuma and Workman, 2008; Cho et al., 2007). In contrast, the crosstalk between the H3K27 methyltransferase EZH2 and the H3K4 demethylase Jarid1a (Pasini et al., 2008) or the euchromatic histone methyltransferase G9a and the H3K4 demethylase Jarid1a promotes gene repression (Chaturvedi et al., 2012).

Euchromatic histone methyltransferase G9a (also known as EHMT2 or KMT1C), along with its close paralogue GLP (also known as EHMT1 or KMT1D), belongs to the SET domain-containing Su(var)3–9 family of proteins (Shinkai and Tachibana, 2011). Both proteins form a heterodimer and regulate mono- and dimethylation of histone H3K9 in euchromatin (Shinkai and Tachibana, 2011). In addition, G9a and GLP are reported to mediate the dimethylation of H3K27 (Chaturvedi et al., 2009; Tachibana et al., 2005; Tachibana et al., 2002), which is a mark of repression. G9a and GLP are essential for mouse development, as knockout of either is embryonic lethal (Tachibana et al., 2005; Tachibana et al., 2002). Functionally, it plays a crucial role in regulating various cellular processes, including cell proliferation, differentiation, senescence, and replication, and functions as both a transcription corepressor and a transcription coactivator, depending on the interacting partners (Poulard et al., 2021). It mediates gene repression by adding mono- and dimethyl lysine 9 on histone 3 (Shinkai and Tachibana, 2011). These methylation marks coordinate with the HP1-dependent repressor complex (El et al., 2008) to mediate gene repression. Additionally, G9 mediates transcriptional repression by collaborating with other corepressors; for example, G9a crosstalks with DNA methyltransferases, viz., DNMT3a/DNMT1, and facilitates gene repression by facilitating DNA methylation (Epsztejn-Litman et al., 2008; Estève et al., 2006). Similarly, it forms a distinct complex with the polycomb repressive complex-2 proteins, including the H3K27 methyltransferase EZH2, to transcriptionally silence specific genomic regions (Mozzetta et al., 2015).

On the contrary, G9a also mediated gene activation, and its coactivator function is independent of its enzymatic activity. Instead, the coactivator function of G9a depends on its interactions with other transcription factors and cofactors involved in gene activation, as reported for nuclear receptor genes and adult *β-globin genes* (Shankar et al., 2013). While G9a interacts with the histone acetyltransferase p300 and the arginine methyltransferase CARM1 to facilitate gene activation of nuclear receptor genes (Lee et al., 2006), it associates with the mediator coactivator complex to facilitate the activation of adult *β-globin genes* (Chaturvedi et al., 2012). However, the exact molecular mechanism underlying G9a-mediated gene activation remains unclear.

In the current study, we demonstrate that G9a crosstalks with H3K36 histone methyltransferases NSD3 (Nuclear SET domain containing protein 3), or WSHC1L1 (Morishita et al., 2014), and SETD2 or KMT3A (Dambacher et al., 2010; Hyun et al., 2017) to mediate its coactivator function. G9a interacts with and recruits NSD3 to the G9a target adult *β-globin* genes, and NSD3 activates the expression of these genes by stabilizing the binding of the coactivator Mediator complex to the promoter and by facilitating SETD2-mediated H3K36 trimethylation in the coding region. Our study reveals a previously undescribed crosstalk mechanism in which G9a mediates gene activation by associating with the two H3K36 histone methyltransferases, NSD3 and SETD2.

## Materials and methods

### Antibodies used for Western blotting (WB)

From Abcam we used Anti-NSD3 (ab180500); Anti-G9a (ab229455); Anti-GLP (ab41969); Anti-NSD1 (orb412944); Anti-NSD2 (ab75359) Anti-Histone H3K36me1 (ab9048); Anti-Histone H3K36me2 (ab9049); Anti-Histone H3K36me3 (ab9050); Anti-Histone H3K9me2 (ab1220); Anti-Histone H3K27me2 (ab24684); Anti-Histone H1 (ab134914); Anti-Histone H3 (ab61251); Anti-Jarid1A (ab 70892) From Santacruz, we used Anti-MED12 (SC-537, SC-192); Anti-Med4 (SC-133779); Anti-RNA Pol-II (SC-900, SC-13583) Anti-TF_II_H (SC-293). We used Anti-Ash2l Ab described previously (Demers et al., 2007); from Cell Signaling Technology, we used Anti-SETD2 (80290).

### For immunoprecipitation (IP), we used the following antibodies

Anti-NSD3 (#11345-1-AP) from Protein Tech, and anti-G9a (PP-A8620A-00) and anti-GLP (PP-B0422-00) from Perseus Proteomics. From Santacruz, we used anti-Med 12 (TRAP 230) (SC-515695), anti-IgG mouse Isotype control (SC-2025). From Abcam, we used anti-IgG rabbit isotype control (ab37415). Anti-SETD2 (same as WB).

### For ChIP, we used the following antibodies

Anti-NSD3 (same as IP); Anti-G9a (same as WB); Anti-RNA Pol II (Merck Millipore 17-620 ChIP Ab), Anti-RNA Pol-II (SC-13583, SC-17798), Anti-MED1 (Merck Millipore 17-10530), Anti-Med 12 (Cell Signaling technology 14360), and Anti-Med17 (SC-12453); Rabbit IgG-Isotype control (ab37415); For Histones ChIPs: H3K9me2, H3K27me2, H3K36me2, H3K36me3 same as WB. Anti-SETD2 (same as WB).

### Cell culture

Murine erythroid (MEL) cells (clone 745), which are blocked at the pro-erythroblast stage and serve as a model system for terminal erythroid differentiation in the definitive lineage, were used to carry out the study. In these cells, treatment with DMSO induces erythroid differentiation, including activating the adult *β-globin* genes and hemoglobin synthesis (Levy et al., 1975; Charnay et al., 1984). Cells were cultured in RPMI-1640 containing 10 mM L-Glutamine (Sigma-Aldrich) supplemented with 10% (vol/vol) FBS (Wisent Inc.), 1% penicillin/streptomycin (vol/vol) (Gibco®), and were differentiated toward erythroid lineage by adding 2% (vol/vol) DMSO for 96 h. Stable doxycycline (dox) dependent MEL single NSD3 knockdown (KD), G9a knockdown, and G9a-NSD3 double knockdown clones generated from MEL, Murine erythroleukemia (745A) cells expressing tetracycline repressor were grown in RPMI-1640 with 10 mM L-Glutamine (Gibco®) supplemented with 10% (vol/vol) Tet-free FBS, (Takara), 20 μg/mL of Blasticidin (Wisent Inc.), 1% penicillin/streptomycin (vol/vol) (Gibco®), G418 Geneticin® (400ug/mL, Cell clone.) and puromycin (200ug/mL). 5 μg/mL doxycycline was used to induce knockdown in these cells. Human K562 cells were grown in RPMI 1640 with 10 mM L-glutamine (Gibco), 10% FBS (vol/vol) (Sigma-Aldrich), and 1% Pen/Strep (vol/vol). Human HEK 293FT cells were grown in D-MEM (high glucose), 10% FBS (vol/vol), 0.1 mM MEM Non-Essential Amino Acids (NEAA), 6 mM L-glutamine, 1 mM MEM sodium pyruvate, 1% Pen-Strep (vol/vol), 500 μg/mL geneticin. MS-5 stromal cells were grown in α-MEM (Gibco), 10% FBS (vol/vol), 6 mM L-glutamine, and 1% Pen/Strep (vol/vol).

### Nuclear extract, immunoprecipitation, and LC-MS/MS

Nuclear extracts and IPs were performed as previously described (Demers et al., 2007). Briefly, for IPs, antibodies (Abs) were crosslinked on Dynabeads™ protein A (for rabbit Abs) and protein G (for mouse and goat Abs) with 20 mM (final concentration) dimethylpimelimidate (Sigma). Nuclear extracts were added to the antibody-crosslinked beads and incubated overnight at 4 °C on a rotating mixer. The proteins bound to protein A/G beads-antibodies were washed twice with IP300 buffer containing 25 mM Tris, pH 7.9, 5 mM MgCl_2_, 10% (v/v) glycerol, 0.1% (v/v) NP40, 0.3 mM DTT, protease inhibitors cocktail (PIC), and 300 mM KCl, then twice with IP 100 buffer containing 100 mM KCl. Antibody-bound proteins were eluted with 6 M urea for 2 h at 37 °C or 6x lamelli buffer at 95 °C for 10 min. A mock IP under the same conditions was done in parallel using normal rabbit IgG or mouse IgG (Santa Cruz Biotechnology). Eluted proteins were then analyzed by LC-MS/MS as described previously (Chaturvedi et al., 2012) or separated on SDS-PAGE for Western blotting. The antibodies used for immunoprecipitation (IPs) and Western blotting are mentioned above.

### Establishment of stable MEL cell lines with inducible knockdown of NSD3 and G9a

MEL cell line stably expressing the Tetracycline Repressor (MEL/TR) was used to obtain a stable doxycycline-inducible single knockdown of NSD3 (Demers et al., 2007). The MEL/TR cells were transfected with the pGJ10-NSD3 vectors. The pGJ10-NSD3 vectors were obtained subsequently by introducing an shRNA sequence targeting NSD3 mRNA 5′-GAT​CCC​GGG​CAA​GAC​AGG​CTT​ATA​ATT​CAA​GAG​ATT​ATA​AGC​CTG​TCT​TGC​C CTTTTTGGAAA-3' (shRNA1) and 5′-GAT​CCC​GAG​AGA​AAG​GGA​AAG​TTA​ATT​C AAG​AGA​TTA​ACT​TTC​CCT​TTC​TCT​CTT​TTT​GGA​AA-3' (shRNA2) under the dependence of a Dox-inducible H1 promoter. The vectors expressing the NSD3 shRNAs were then transfected separately into the MEL/tetracycline repressor (TetR) cell line, which expresses high levels of TetR. After screening for G418-resistant cells, stable clones that display a Doxycycline-Inducible knockdown (KD) of NSD3 were selected using Western blot and RT-qPCR. The knockdown was induced using 5 μg/mL Dox. For the G9a knockdown, a previously described Dox-inducible G9a knockdown clone expressing G9a shRNA (5′CCC​TGA​TCT​TTG​AGT​GTA​A-3 ′) was used (Chaturvedi et al., 2009). To obtain MEL/TR clones with double knockdown (KD) of G9a plus NSD3, the previously described G418-resistant G9a KD MEL clone was transfected with the pGJ10-puro-NSD3sh1 vector. MEL/TR cells resistant to G418 and Puromycin were selected and screened by Western blot and RT-qPCR for efficient Dox-inducible double knockdown of G9a and NSD3.

### Knockdown of SETD2

SETD2 knockdown was established in the MEL cell line by transfecting Dharmacon ON-Target plus siRNA for SETD2 (J-062392-09-0050) and negative control (001810-10-50) using RNAi-MAX transfection reagent (Invitrogen) according to the manufacturer’s instructions.

### Benzidine staining

1 × 10^6^ cells were incubated in the benzidine solution containing 0.4% Benzidine dihydrochloride in 12% glacial acetic acid and 0.3% H_2_O_2_ (added to the solution before use) for 5 min at RT. The benzidine-positive cells were counted, and the images were captured using a phase-contrast microscope.

### Growth curve

0.3 × 10^6^ cells/mL were cultured in 100 mL of culture media containing 2% DMSO. Cells were counted every 24 h up to 96 h. At each time point, 2 × 10^6 cells were pelleted, RNA isolated, and subsequently analyzed by RT-qPCR.

### RNA extraction and qPCR analysis

Total RNA was isolated using the Trizol Reagent (Ambion Life Technologies) according to the manufacturer’s instructions. Two micrograms of RNA were converted to cDNA using the High-Capacity cDNA Reverse Transcription Kit (Applied Biosystems). Real-time quantitative PCR was performed on a BioRad CFX 96 instrument using SyBr Green or minor groove binder TaqMan probes (ABI) and gene-specific primers (sequences available in Table 1). GAPDH was used for normalization, and relative quantification was done as described previously (Chaturvedi et al., 2012). Three independent biological replicates were used for statistical analysis, and standard deviations were calculated to generate error bars. Statistical significance was determined using a Student *t-test*. The primers and probes used for qRT-PCR are mentioned in Table 1.

**TABLE 1:** List of TaqMan probes and primers and SYBR Green primers used in real-time quantitative PCR.

S.No	Name	Sequence
1	HS2	For: 5′CAGAGGAGGTTAGCTGGGCC-3′ Rev: 5′CAAGGCTGAACACACCCACA-3′ Probe: FAM-AGGCGGAGTCAATTCTCTACTCCCCACC-BHQ1
2	E^Y^ promoter	For: 5′CTTCAAAGAATAATGCAGAACAAAGG-3′ Rev: 5′CAGGAGTGTCAGAAGCAAGTACGT-3′ Probe FAMATTGTCTGCGAAGAATAAAAGGCCACCA CTT-BHQ1
3	E^Y^ ex2	For: 5′GCAAGAAGGTGCTGACTGCTT-3′ Rev: 5′GTAGCTTGTCACAGTGCAGTTCACT-3′ Probe: FAMTGGAGAGTCCATTAAGAACCTAGACAACCTCAAGTC–MGBNFQ
4	ß^major^ prom	For: 5′CTGCTCACACAGGATAGAGAGGG-3′ Rev: 5′GCAAATGTGAGGAGCAACTGATC-3′ Probe: FAM-AGCCAGGGCAGAGCATATAAGGTGAGGT-BHQ1
5	ß^major^ ex2	For: 5′GAAGGCCCATGGCAAGAAG-3′ Rev: 5′GCCCTTGAGGCTGTCCAA-3′ Probe: FAM-TGATAACTGCCTTTAACGATGGCCTGAA TCA-MGBNFQ
6	ß^major^ ex3	For: 5′TCTACAGTTATGTTGATGGTTCTTCCA-3′ Rev: 5′CAGGACAATCACGATCATATTGC-3′ Probe: FAM- TCCCACAGCTCCTG-MGBNFQ
7	ß^minor^ ex3	For: 5′GCAATGCGATCGTGATTGTG-3 Rev: 5′CAGCCACCACCTTCTGGAA-3′ Probe: FAM- CCCCTGCTGCACAGG-MGBNFQ
8	G9a	For: 5′AAAACCATGTCCAAACCTAGCAA-3′ Rev: 5′GCGGAAATGCTGGACTTCAG-3′ Probe:5′FAM-ACAGCCTCCAATCCCTGAGAAGCGG-BHQ1-3′
9	GLP	For: 5′TGTGTGACATCCCCCATGAA-3′ Rev: 5′GCAGTCATCTACACACACGCAGTA-3′ Probe: 5′ FAM-CAGGAACATCACTCATT-BHQ1-3′
10	NSD1	For: 5′AGCCACTTAATGGGTGTACTATG-3′ Rev: 3′GTCTCCGCAGTGGAATGTAA-3
11	NSD2	For: 5′ATGTCAATAGAGGAGCGGAAA-3′ Rev: 5′TAACAATGCCAGCCTCCTTAG-3′
12	NSD3	For: 5′ACTCGGGAGGAAAGAGTAGAG-3′ Rev: 5′GGGTTTCTTGACTTCCGTCTTA-3
13	PBGD	For: 5′CGCATACAGACCGACACT-3′ Rev: CAGGCTCTTCTCTCCAATCT-3′
14	FECH	For: 5′CCCTTGGAGAAGTTCAAGAC-3′ Rev: 5′CGATTCTGCGATACTGCTCT-3′
15	GATA1	For: 5′GCACTCTACCCTGCCTCAACTG-3′ Rev: 5′GTGTTGTTGCTCTTCCCTTCCT-3′
16	FOG1	For: 5′CTGCCAGAAAGTGCATCCAA-3′ Rev: CGAAGACTCAGCAAGAACAAGG-3′
17	KLF1	For: 5′CCCCCTTCCTTCTTGAATTGTC-5′ Rev: 5′GCAGTGGCCCCGAGTTC-3′
18	NF-E2	For: 5′TGAGACACCCTTGGCCTTAGA-3′ Rev: 5′CACGGACAGCAGGCTTAGC-3′
19	ALAS1	For: 5′CCCGATGGCGGATGACT-3′ Rev: 5′CACCAGACCGACACCTGCT-3′
20	ALAS2	For: 5′ACGCTGCAGGCTTCATCTTT-3′ Rev: 5′GAGCCCCAGAGAGCACCAT-3′
21	SETD2	For: 5′GGTGTTGCTTCAAGTCGATTTTT-3′ Rev: 5′CGTCCCTGTTCCTCCAAATTAA -3′
22	GAPDH	For: 5′TTGTGGAAGGGCTCATGA -3′ Rev: 5′CATCACGCCACAGCTTT-3′

FAM, is the fluorophore; BHQ, black hole quencher; MGB, minor groove binder; NFQ, non fluorescent quencher.

### Western blotting (WB)

Thirty micrograms of nuclear protein (for knockdown detection) or 1/20 vol of the IPs pulldown samples (for verification of interacting proteins) were loaded and separated by SDS-PAGE. After electrophoresis, the proteins were transferred to a nitrocellulose membrane (BioRad). The membrane was blocked with 5% non-fat dry milk (NFDM) in TBST Buffer containing 10 mM Tris-Cl, pH 7.4,100 mM NaCl, 0.1% (v/v) TWEEN 20 for 1 h at room temperature and subsequently incubated with a primary antibody with 5% NFDM in TBST Buffer at 4 °C overnight (List of antibodies used for WB above in antibodies section). Primary antibodies were detected with corresponding horseradish peroxidase (HRP)-conjugated secondary antibodies (1:5,000). Blot images were captured using an ImageQuant LAS 500 GE Healthcare Chemiluminescence system, and relative intensity was estimated using ImageJ software.

### Histone acid extract

Histone acid extract was prepared by resuspending 1 × 10^6^ differentiated MEL cells in Triton extraction buffer (TEB) containing PBS, supplemented with 0.5% Triton X-100, 2 mM PMSF, and 0.02% NaN3, and lysed on ice for 10 min. Lysed cells were centrifuged at 2000 RPM for 10 min at 4 °C, and the pellet was washed in half the volume of TEB and resuspended in 0.2N HCl at a cell density of 5 × 10^7^ cells/mL. Histones were extracted overnight on the rotator at 4 °C. Five micrograms of histone protein per sample were loaded and separated by SDS-PAGE and then subjected to Western blotting as described above.

### Chromatin immunoprecipitation

Crosslinked ChIPs (X-ChIPs) were performed as described previously (Demers et al., 2007). Briefly, 3-4 ug antibodies were coupled to Dynabeads™ protein A (for rabbit antibodies) or with Dynabeads™ protein G (for mouse and goat antibodies) for 2 h at room temperature in IP 100 buffer containing 25 mM Tris, pH 7.9, 5 mM MgCl_2_, 10% (v/v) glycerol, 0.1% (v/v) NP40, 0.3 mM DTT, proteases inhibitors cocktail and 100 mM KCL. Bead-bound antibodies were washed twice with IP 100 buffer and used for immunoprecipitation. For chromatin extraction, 50 × 10^6^ cells were crosslinked for 20 min at room temperature with 1% (w/w) final formaldehyde, and the reaction was stopped with 125 mM glycine. Nuclei were isolated by resuspending and incubating cells in a swelling buffer containing 25 mM HEPES (pH 7.9), 1.5 mM MgCl2, 10 mM KCl, 0.1% NP-40, 1 mM DTT, and PIC on Ice for 15 min. Subsequently, cells were lysed using a Dounce homogenizer Type B pestle to obtain nuclei. The isolated nuclei were resuspended in lysis buffer containing 50 mM HEPES (pH 7.9), 140 mM NaCl, 1 mM EDTA, 1% Triton-X 100, 0.1% Na-deoxycholate, 1% SDS, and PIC, and then sonicated using a Diagenode Bioruptor Sonicator at 4 °C with 30 s on and 90 s off for 30 cycles. Sonicated samples were diluted with sonication buffer lacking SDS, resulting in a final SDS concentration of 0.1%. The samples were immunoprecipitated overnight at 4 °C with antibodies coupled to Dynabeads™ protein A/G beads. Immunoprecipitates were washed twice with sonication buffer with 0.1% SDS, then twice with wash buffer A (50 mM HEPES (pH7.9), 140 mM NaCl, 1 mM EDTA, 1% Triton-X-100, 0.1% Na-deoxycholate, 0.1% SDS and PIC) and wash buffer B (20 mM Tris (pH8.0), 1 mM EDTA, 250 mM LiCl, 0.5% NP-40, 0.5% Na-deoxycholate and PIC) and finally twice with TE buffer. After RNase treatment, reverse crosslinking, and Proteinase K treatment, the DNA was purified using the standard phenol/chloroform and ethanol precipitation method. The ChIP DNA was subjected to real-time quantitative PCR on a BioRad CFX 96 instrument using Taq-Man probes (ABI) and primers. ChIP signals are represented as a percentage of input. Statistical significance was determined using a Student t-test. The antibodies used for ChIP are mentioned in the antibodies section above. The primers and Taq-Man probes used are listed in Table 1.

For histone ChIPs, we used a native ChIP protocol (Brand et al., 2008). Briefly, 50 × 10^6^ cells were lysed in buffer N (15 mM Tris (pH 7.5), 15 mM NaCl, 60 mM KCl, 250 mM sucrose, 5 mM MgCl2, 1.5 mM CaCl_2_, 1 mM DTT, 0.6% NP-40, and PIC). Nuclei were recovered by centrifugation and quantified by measuring total DNA content. Chromatin was digested with 1U MNase/5ug chromatin (Sigma) for 10 min at 37 °C to give a maximum visible fragment size of 150–300 base pairs (one to two nucleosomes). NaCl was then added to a final concentration of 600 mM, and the nuclei were incubated with 10 mg of hydroxyapatite resin to make a slurry. After extensive washing, nucleosomes were eluted from the resin with a buffer containing 300 mM NaPO_4_ (pH 7.2). Eluted nucleosomes were subjected to Immunoprecipitation with either anti-histones or control rabbit/mouse IgG. As mentioned above, Immunoprecipitation, DNA purification, and PCR analysis were done as for cross-linked ChIPs. The primers and probes used for qRT-PCR are mentioned in Table 1.

### G9a-NSD3 direct interaction

The G9a-NSD3 direct interaction was performed as previously described (Chaturvedi et al., 2009). Briefly, equivalent amounts of Recombinant purified UBC4 (Boston Biochem, Cat no. U-120), G9a (Active Motif, Cat. No. 31410), WHSC1L1/NSD3-SET protein (Active Motif, Cat No. 31477) proteins were incubated on ice in the IP-100 buffer containing 25 mM Tris, pH 7.9, 5 mM MgCl_2_, 10% (v/v) glycerol, 0.1% (v/v) NP40, 0.3 mM DTT, proteases inhibitors cocktail and 100 mM KCL for 1 h prior to Immunoprecipitation with the G9a Ab-coupled Dynabeads™ for 2 h at 22 °C with constant shaking at 1400 RPM followed by extensive washing with IP-100 buffer. The bound proteins were eluted in 6X Laemmli buffer at 95 °C for 10 min, and the samples were analyzed by Western blotting.

### Size exclusion chromatography

Nuclear extracts from MEL and K562 cells were fractionated by size-exclusion chromatography using a Superose 6 10/300 GL column on an AKTA explorer HPLC system, according to the manufacturer’s instructions (Amersham). Fractions of 0.5 mL were collected, precipitated with 10% trichloroacetic acid (TCA), and analyzed by Western blot.

### Sequential chromatin immunoprecipitation (ReChIP)

To investigate the co-occupancy of G9a and NSD3 at the β-globin loci, we employed a sequential chromatin immunoprecipitation (ReChIP) technique, following our established ChIP protocol. Chromatin was prepared and subjected to immunoprecipitation (IP1) using an antibody targeting G9a. The DNA-protein complexes bound to the beads were then eluted using a first ChIP elution buffer (50 mM Tris-HCl, pH 8.0, 5 mM EDTA, 20 mM DTT, and 1% SDS) and incubated at 65 °C for 10 min with gentle agitation. The eluate was purified using 10 mg of Hydroxyapatite resin. For the second round of immunoprecipitation, the diluted eluate was incubated overnight at 4 °C with antibodies specific to G9a (IP2.1) or NSD3 (IP2.2). The beads were sequentially washed with low-salt, high-salt, LiCl, and TE buffers. The DNA-protein complexes were eluted from the beads with the elution buffer and incubated at 65 °C for 10 min. To reverse the cross-links, NaCl was added to the combined eluates to a final concentration of 200 mM, followed by incubation at 65 °C for 45 min and Proteinase K treatment. The DNA was purified using the standard phenol/chloroform and ethanol precipitation method. The ChIP DNA was subjected to real-time quantitative PCR on a BioRad CFX 96 instrument using Taq-Man probes (ABI) and primers. ChIP signals are represented as a percentage of input. Statistical significance was determined using a Student t-test. The antibodies used for ChIP are mentioned in the antibodies section above. The primers and Taq-Man probes used are listed in Table 1.

## Results

### NSD3 is a subunit of the G9a coactivator complex

**FIGURE 1:**
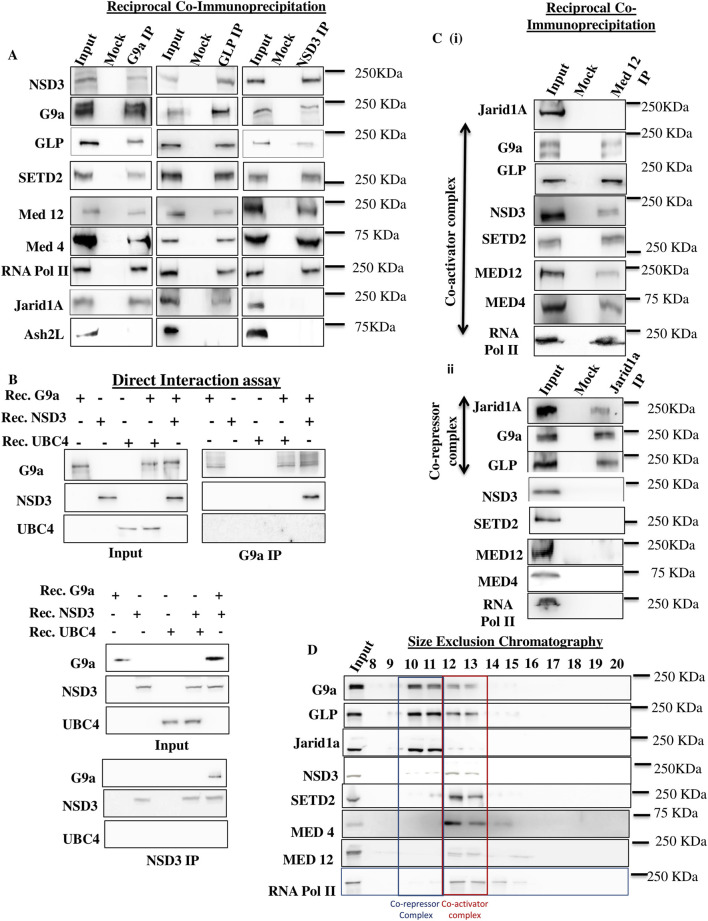
NSD3 and SETD2 are part of the G9a coactivator complex. **(A)** Proteins extracted from MEL differentiated cells were immunoprecipitated using antibodies against G9a, GLP, and NSD3 and analyzed by Western blot. A mock IP with normal IgG was used as a negative control. Antibodies used for Western blot (Left) and molecular masses (Right; in kilodaltons) are indicated. **(B)** NSD3 interacts directly with G9a. Rec purified NSD3 (a.a. 1,021-1,320) or Ubc4 was incubated with rec-purified G9a before IP with G9a or NSD3 Abs. Immunoprecipitated proteins were detected by immunoblotting using the antibodies indicated on the left. **(C)** NSD3 co-elutes with the G9a-GLP complex, Mediator Complex, and RNA Pol II, as revealed by size-exclusion chromatography. A nuclear extract was separated on a Superose-6 column, and the presence of specific proteins in different fractions was revealed by immunoblotting with Abs indicated on the left. Molecular masses (in kilodaltons) are shown on the right. **(D)** Proteins extracted from MEL differentiated cells immunoprecipitated via Abs against Med12 and Jarid1A were analyzed by Western blot. A mock IP with normal IgG was used as a negative control. Antibodies used for Western blot (Left) and molecular masses (Right; in kilodaltons) are indicated.

The histone methyltransferase G9a plays a dual functional role in erythroid cell differentiation (Chaturvedi et al., 2009). We have previously shown that the dual function of G9a depends on its ability to form two distinct protein complexes: a corepressor complex and a coactivator complex (Chaturvedi et al., 2012). In the proteomic screen, we identified the nuclear SET domain-containing H3K36 histone methyltransferase NSD3 as an interacting partner of G9a (identification score 99%). Therefore, to confirm whether NSD3 is part of the G9a coactivator complex, we first validated the interaction between NSD3 and the G9a/GLP complex. Nuclear extract from differentiated erythroid cells (MEL cell line) was used in reciprocal immunoprecipitation using antibodies (Abs) recognizing the endogenous G9a, GLP, and NSD3 proteins. Analysis of immunoprecipitated proteins through Western blotting revealed G9a-GLP were present in NSD3 IP, and NSD3 was found in immunoprecipitations of both G9a and GLP confirming the interaction between the three proteins (Figure 1A). Since the above results were obtained using a mouse MEL cell line, we wanted to determine whether the interaction between G9a and NSD3 is specific to only MEL cells or conserved among other cell types. To address this question, we performed G9a and NSD3 immunoprecipitations (IPs) in nuclear extracts prepared from three different cell lines: K562, a human erythroleukemia cell line (Andersson et al., 1979); 293 T, a human cell line (Lin et al., 2014); and MS5, a mouse stromal cell line (Berthier et al., 1997). Immunoprecipitated proteins were analyzed by Western blotting using specific antibodies against G9a, GLP, and NSD3. The results revealed that the association between G9a and NSD3 is conserved in all 3 cell types, suggesting the interaction between G9a and NSD3 is conserved in cells of both human and mouse origin (Supplementary Figure S1). Furthermore, to investigate the nature of the interaction between G9a and NSD3, we incubated recombinant, purified G9a and NSD3 proteins and performed immunoprecipitations (IPs) for G9a and NSD3. As shown in Figure 1B, rec G9a specifically pulled down NSD3 but not the control protein (rec UBC-4), demonstrating a direct interaction between G9a and NSD3.

We next investigated whether NSD3 is part of the G9a coactivator complex. We performed Western blotting for the coactivator Mediator complex (Med12 and Med4 subunits), RNA Pol II, and corepressor Jarid1a on G9a, GLP, and NSD3 IP pulldown samples. Consistent with our previous finding (Chaturvedi et al., 2012) and as evident in Figure 1A, Mediator complex (Med12 and Med4 subunits), RNA Pol II, and Jarid1a immunoprecipitated with G9a and GLP, suggesting that the G9a/GLP complex interacts with both coactivator and corepressor proteins. In contrast, the NSD3 IP showed the association of NSD3 with G9a, GLP, the mediator complex (Med12 and Med4 subunits), and RNA Pol II, but not with the corepressor Jarid1 (Figure 1A). Furthermore, reciprocal IPs using coactivator Med12 Abs also confirmed Med12 interaction specifically with G9a, GLP, Med4, RNA Pol II, and NSD3 but not with Jarid1a, while Jarid1a IP showed interaction with G9a, GLP but not with mediators, RNA Pol II, and NSD3 (Figure 1C (i-ii)). These results proved that NSD3 physically interacts with G9a and is part of the G9a coactivator complex.

To investigate whether NSD3 associates with the G9a-GLP activator complex in the cells, we performed size exclusion chromatography on nuclear extracts from differentiated MEL cells. G9a and GLP are part of a heterodimer, and these two proteins co-elute in the same gel filtration fractions (Figure 1D, fractions 10 to 13). Interestingly, out of the four gel fractions, the first two fractions of the G9a-GLP complex predominantly co-eluted with the peak of corepressor Jarid1a (Figure 1D, fractions 10 and 11). Meanwhile, the third and fourth fractions of the G9a-GLP complex peak with NSD3, the coactivator Mediator complex, and RNA Pol II fractions (Figure 1D, fractions 12–13). These results highlight that the G9a-GLP complex exists in two distinct complexes *in vivo*, as described previously (Chaturvedi et al., 2012), one, the corepressor complex, having G9a-GLP complex associated with corepressor Jarid1a, and the other, coactivator complex, where the G9a-GLP complex associated with NSD3, Mediator complex, and RNA Pol II, thus validating the above IP results.

### NSD3 regulates H3K36 dimethylation in differentiated erythroid cells

**FIGURE 2:**
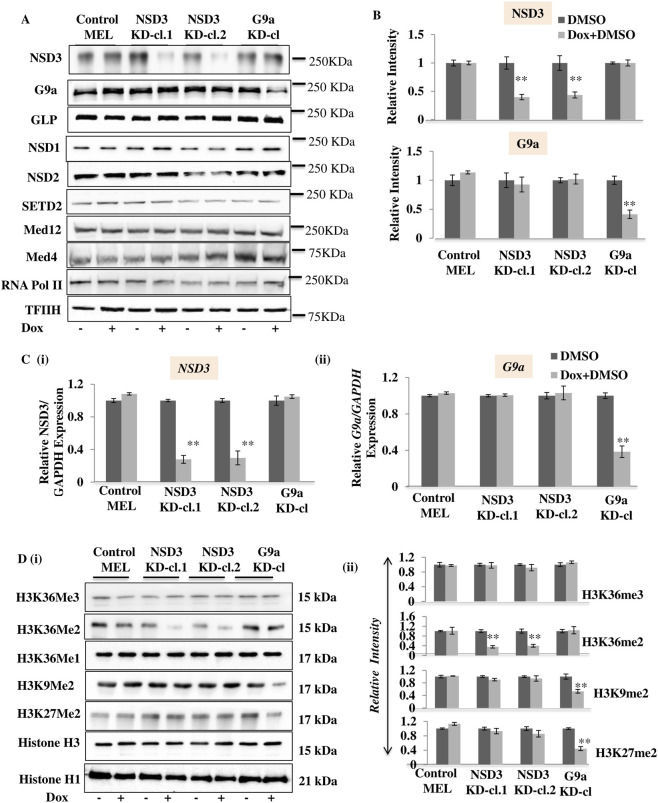
Establishment and characterization of Dox inducible MEL cell lines with NSD3 knockdown (KD). This figure shows the results using NSD3 shRNA1, NSD3 shRNA2, and previously reported G9a knockdown (KD) cl1 clones. **(A)** The levels of NSD3 and G9a in Dox-treated (Dox) vs. untreated (No Dox) cells were analyzed at the protein level by Western blot of nuclear extracts using the Abs indicated (Left) and molecular masses (in kilodaltons) are indicated (Right), **(B)** along with the relative intensity of the Western blots and **(C)** (i), (ii) at the mRNA level by real-time RT-qPCR. MEL parent represents a control cell line with no shRNA induction upon Dox treatment. Transcript levels are normalized to GAPDH with the ratio observed in the absence of Dox set to 1. **(D)** (i) Bulk cellular levels of H3K36Me2, H3K36Me3, H3K9Me2, H3K27Me2, and other histone modifications were analyzed by Western blot before and after NSD3 and G9a KD as indicated. (ii) Relative enrichment of H3K36Me3, H3K36Me2, H3K27Me2, and H3K9Me2 analyzed by Western blot was quantified using ImageJ. Error bars represent SDs calculated from triplicate experiments. *P < 0.05; **P < 0.01.

To elucidate the functional significance of NSD3 in differentiated erythroid cells and G9a-mediated gene activation, we developed a stable mouse erythroleukemia (MEL) cell line that, upon doxycycline treatment, can induce stable knockdown (KD) of NSD3. We used RNA interference to KD the NSD3 protein in differentiated adult erythroid cells. Two clonal mouse erythroleukemia (MEL) cell lines expressing doxycycline (Dox) inducible small hairpin shRNA sequences were generated against regions targeting the NSD3 coding region and 5′UTR region. In both clones, dox treatment resulted in a significant decrease (between 60% and 70%) in the expression of NSD3 at both the transcript and protein levels, whereas NSD3 knockdown did not affect the expression of G9a and GLP (Figures 2A–C). As NSD3 interacts with G9a, we also investigated whether G9a knockdown affects NSD3 levels using a previously published G9a KD cl1 (11). Dox-induced knockdown of G9a resulted in a significant decrease of G9a, as previously reported (Chaturvedi et al., 2009), without affecting the level of NSD3 (Figures 2A–C). These results suggest that NSD3 knockdown did not affect G9a and GLP levels and *vice versa*. Subsequently, we investigated the effect of NSD3 knockdown on bulk histones using Western blot analysis. Consistent with previous studies (Jeong et al., 2020), depletion of NSD3 leads to a significant decrease of bulk level of H3K36me2 without changing the levels of H3K36me1/3 or G9a mediated H3K9me2 and H3K27me2 marks (Figure 2D (i-ii)). Similarly, G9a knockdown does not affect the bulk levels of H3K36me3, H3K36me2, and H3K36me1, whereas H3K9me2 and H3K27me2 levels were reduced, as reported earlier (Chaturvedi et al., 2009) (Figure 2D (i-ii)). Furthermore, we analyzed the effects of G9a or NSD3 knockdown on GLP and other H3K36 methyltransferases, including NSD1, NSD2, and SETD2, and found no change in their protein levels (Figure 2A; Supplementary Figure S2). These findings suggest that NSD3 specifically mediates H3K36me2 in the differentiated erythroid cells.

### NSD3 activates G9a target adult *β* ^*major*^ *and β* ^*minor*^ *globin* genes in differentiated erythroid cells

**FIGURE 3:**
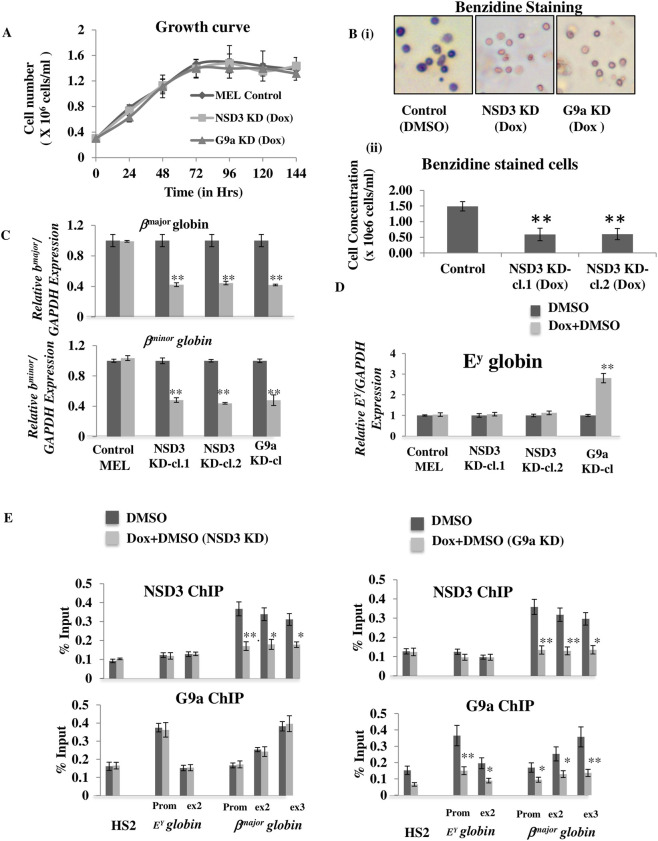
NSD3 regulates the activation of G9a target *adult β* ^*major*^ *and β* ^*minor*^ *globin* genes. **(A)** NSD3 or G9a single KD does not affect cell growth. Cell concentration was assessed every 24 h after induction of erythroid differentiation in NSD3 or G9a depleted (Dox) vs. normal (No Dox) MEL cells. **(B)** (i) NSD3 or G9a KD decreases hemoglobinization to comparable levels in erythroid cells. Hemoglobin content was assessed by benzidine staining in NSD3 or G9a depleted (Dox) vs. normal (No Dox) MEL cells after 96 h of erythroid differentiation. **(B)** (ii) Quantitative representation of benzidine positive cells in NSD3 or G9a depleted cells vs. Control MEL cells in differentiated condition. **(C)** Transcription of *β* ^*major*^ *globin*, *β* ^*minor*^ *globin, and* **(D)** *E* ^*Y*^ *globin* genes were assessed by RT-qPCR after differentiation in NSD3 or G9a depleted (Dox) vs. normal (No Dox) MEL cells. Transcripts are expressed relative to GAPDH with the highest ratio set to 1. **(E)** NSD3 and G9a ChIPs were performed after differentiation in NSD3 or G9a depleted (Diff. NSD3/G9a KD) vs. normal (Diff.) MEL cells to analyze the binding of NSD3 or G9a enrichment on LCR HS2, repressed *E* ^*Y*^ *globin,* and active *β* ^*major*^ *globin* genes. The ChIP DNA was analyzed by quantitative PCR (qPCR) with specific primers and probes. ChIPs were revealed by real-time qPCR using specific primers and probes located at the HS2 site of the locus control region, promoter, and coding regions of *E* ^*Y*^ and *β* ^*major*^ *globin* genes. Average values from triplicate experiments are represented with error bars corresponding to the standard deviations (SDs). P*<0.05; **P < 0.01.

The G9a coactivator complex activates adult *β* ^*major*^ *and β* ^*minor*^ *globin* gene expression in differentiated erythroid cells (Chaturvedi et al., 2012). As we identified NSD3 as part of the G9a coactivator complex, we speculated that NSD3’s interaction with G9a is essential for activating G9a-targeted adult *β-globin* genes. Therefore, we evaluated the effect of NSD3 knockdown on cell growth, hemoglobinization, the expression of G9a target active adult *β* ^*major*^ *and β* ^*minor*^ *globin* genes, and the repression of the embryonic *E* ^*Y*^ *globin* gene in differentiated erythroid cells. While the knockdown of NSD3 did not affect the proliferation of MEL cells, a significant decrease in hemoglobinization was observed in differentiated MEL cells, consistent with the phenotype observed upon G9a knockdown (Figures 3A–C). To test whether this defect in hemoglobinization is due to deregulation of *β-globin* genes, we performed RT-qPCR. While knockdown of G9a resulted in decreased expression of adult *β* ^*major*^ *and β* ^*minor*^ *globin genes* concomitant with reactivation of *E* ^*Y*^ *globin gene* as previously described (Chaturvedi et al., 2009) and shown in Figure 3D, the NSD3 knockdown has no effect on G9a repressed embryonic *E* ^*y*^ *globin* gene*;* however, a significant reduction in adult *β* ^*major*^ *and β* ^*minor*^ *globin* genes expression was observed upon NSD3 KD (Figures 3C,D).

Previously Microarray analysis of differentiated MEL cells vs. G9a KD MEL cells has shown no alterations in the expression of most of the genes, including the genes involved in heme biosynthesis *(FECH, PBGD, ALAS1* and *ALAS2)* and erythroid differentiation (*GATA1, EKLF, FOG1,* and *NF-E2) genes,* suggesting that G9a exclusively regulates erythroid differentiation by regulating adult *β* ^*major*^ *and β* ^*minor*^ *globin* genes (Chaturvedi et al., 2009). Consistent with these results, the knockdown of NSD3 also showed no effect on the expression of all these genes (Supplementary Figure S3), highlighting that the loss of NSD3 specifically regulates the expression of G9a target adult *β-globin* genes in differentiated erythroid cells without affecting the heme biosynthesis *and* other erythroid differentiation associated genes (Supplementary Figure S3).

While both G9a and NSD3 depletion repressed the adult *β* ^*major*^ and *β* ^*mino*^ *r globin* genes, we next asked whether the combination of G9a and NSD3 KD additively suppresses the expression of the adult *β globin* gene more than their single knockdowns. To evaluate this possibility, we generated a double knockdown MEL cell clone that expressed inducible shRNAs targeting both G9a and NSD3. Dox treatment of double knockdown clones results in a significant decrease in the expression of NSD3 and G9a at both the transcript and protein levels, which was comparable to the single knockdown of NSD3 or G9a (Supplementary Figure S4A,B). The double knockdown exhibited reduced hemoglobinization, accompanied by decreased expression of *the β* ^*major*^ and *β* ^*minor*^ *globin genes,* comparable to the single knockdown of either NSD3 or G9a, indicating no significant additive effect (Supplementary Figures S4A,C,D). These results suggest that while a single knockdown of either G9a or NSD3 is sufficient to downregulate the expression of *β* ^*major*^ and *β* ^*minor*^ *globin* genes, depleting both proteins does not have a significant additive effect or stronger silencing of *β* ^*major*^ and *β* ^*minor*^ *globin* genes compared to single knockdown (Supplementary Figure S5D). Furthermore, as expected, the repressed *E* ^*Y*^ *globin* gene remains unchanged upon NSD3 knockdown, whereas G9a knockdown leads to reactivation comparable to double knockdown (Supplementary Figure S4D).

### NSD3 is recruited to the *β* ^*major*^ *globin* gene in a G9a-dependent manner

**FIGURE 4:**
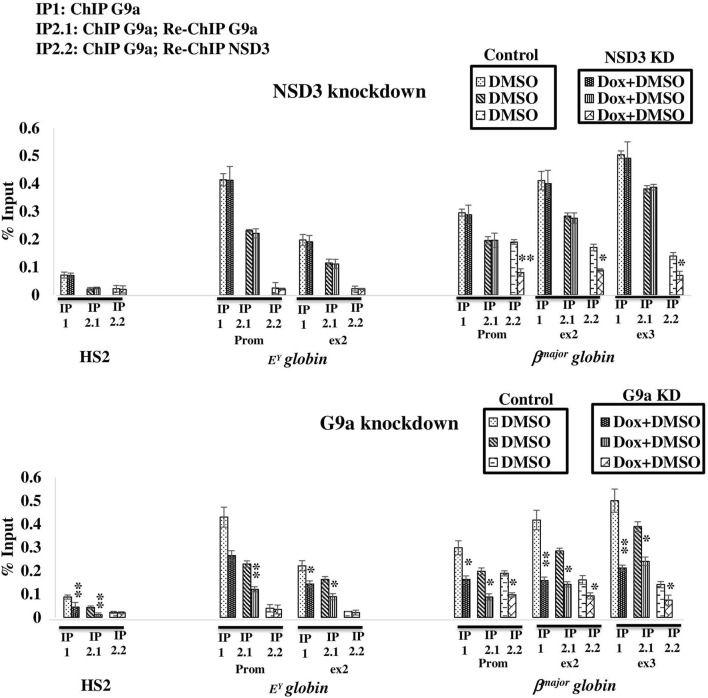
NSD3 is recruited to *β-globin* locus in a G9a dependent manner. ChIP and ReChIP experiments were performed after induction of erythroid differentiation in NSD3-or G9a-depleted (Diff. NSD3/G9a KD) cells versus normal (Diff.) cells. MEL cells were used to analyze the binding of G9a (IP1), while ReChIP of the elution product of IP1 was used to analyze the binding of G9a (IP2.1) and NSD3(IP2.2). Binding and localization of G9a and NSD3 in ChIPs were revealed by real-time qPCR using specific primers and probes located at the HS2 site of the locus control region and promoter of *E* ^*Y*^ *globin* and *β* ^*major*^ *globin genes*. Error bars represent SDs calculated from triplicate experiments. *P < 0.05; **P < 0.01.

To determine whether NSD3 binds to the *β* ^*major*^ *globin* gene to regulate its expression, we performed NSD3 ChIP experiments in differentiated cells with NSD3 knockdown. ChIP revealed that NSD3 is localized on the active *β* ^*major*^ *globin* gene, predominantly on the promoter region with a decrease towards the 3′ coding region (Figure 3E). A significant decrease in the ChIP signals was observed upon NSD3 knockdown, demonstrating the specificity of the NSD3 antibody used. No binding of NSD3 was detected in the HS2 and repressed *E* ^*Y*^ *globin* gene (Figure 3E). Previously, it was shown that G9a is bound on the entire *β* globin locus, including the repressed embryonic *E* ^*Y*^ and active *β* ^*major*^ *globin* genes (Chaturvedi et al., 2009). Consistent with this, G9a binding was detected on LCR-HS2, repressed *E* ^*Y*^ *globin* gene, and functional *β* ^*major*^ *globin* gene, and a significant reduction in G9a binding was observed upon G9a knockdown (Figure 3E). Next, we asked whether the recruitment and binding of NSD3 to the active *β* ^*major*^ *globin* gene depend on G9a. To study this, we performed ChIP assays for G9a and NSD3 in differentiated cells with G9a knockdown. Interestingly, NSD3 binding to the active *β* ^*major*^ *globin gene* also decreased upon G9a KD (Figure 3E), highlighting that G9a is necessary for NSD3 binding to this gene. In contrast, we observed that NSD3 knockdown does not affect the binding of G9a, suggesting that G9a binding to the *β* ^*major*^ *globin* gene is independent of NSD3 (Figure 3E). Furthermore, to validate the same, we performed ChIP of G9a (IP1) followed by ReChIP of G9a (IP2.1) or NSD3 (IP2.2) in NSD3 or G9a knockdown MEL cell line (Figure 4), we observed that ReChIP of G9a showed similar pattern of G9a enrichment as evident in G9a ChIP (Figure 3E) in both NSD3 and G9a knockdown MEL cell line. Likewise, ReChIP of NSD3 (IP2.2) showed a similar pattern of NSD3 enrichment on *β-globin* locus as evident in NSD3 ChIP in both NSD3 and G9a knockdown MEL cell line (Figures 3E, 4). These results demonstrate that NSD3 is recruited and localized to the active *β* ^*major*^ *globin* gene in a G9a-dependent manner.

### NSD3 activates the G9a target *β* ^*major*^ *globin* gene by stabilizing the mediator complex on the promoter region

**FIGURE 5:**
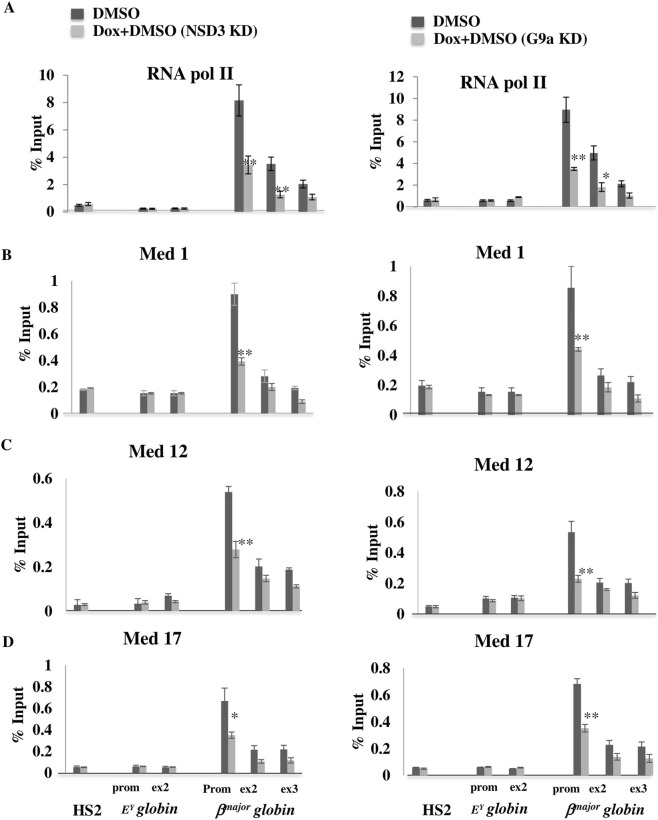
NSD3 is vital for associating the Mediator complex and RNA polymerase II with the G9a to form a coactivator complex. NSD3 stabilizes the binding of the RNA Pol II and Mediator complex to the G9a target active *β* ^*major*^ *globin* gene promoter. ChIP experiments were performed after induction of erythroid differentiation in NSD3 or G9a depleted (Diff. NSD3/G9a KD) vs. normal (Diff.) MEL cells to analyze the binding of RNA Pol II **(A)**, Med 1 **(B)**, Med12 **(C)**, and Med17 **(D)** subunit of the Mediator complex. ChIPs were revealed by real-time qPCR using specific primers and probes located at the HS2 site of the locus control region and promoter of *E* ^*Y*^ *globin* and *β* ^*major*^ *globin genes*. Error bars represent SDs calculated from triplicate experiments. *P < 0.05; **P < 0.01. Depleting NSD3 disrupts the interaction between G9a and the Mediator complex/RNA Polymerase II.

G9a mediates the transcriptional activation of adult *β-globin genes* by stabilizing the binding of pre-initiation complex (PIC) components, RNA Polymerase II, and the Mediator complex to the promoter region (Chaturvedi et al., 2012; Chaturvedi et al., 2009). Since NSD3 interacts with and colocalizes with RNA Pol II and the mediator complex, as revealed by IP and gel filtration, respectively (Figures 1A,C), we first tested whether NSD3 knockdown also alters RNA Pol II binding to the *β* ^*major*^ *globin* gene, as observed with G9a knockdown. ChIP was done to examine the binding of this enzyme after NSD3 and G9a knockdown. Consistent with the G9a knockdown, NSD3 knockdown also leads to a reduction of Pol II binding to the promoter region of this gene (Figure 5A), suggesting that NSD3 stabilizes the binding of RNA Pol II on *β* ^*major*^ *globin* gene, and *a* decrease in NSD3 level downregulates RNA Pol II localization on *β* ^*major*^ *globin* gene, thus leading to a decrease in gene transcription. G9a has been shown to stabilize the coactivator mediator complex binding on this gene in differentiated erythroid cells (Chaturvedi et al., 2012). Hence, we investigated the role of NSD3 in stabilizing the coactivator mediator complex binding to the active *β* ^*major*^ *globin* gene using ChIP in G9a- and NSD3-knockdown cells. Consistent with our previous study (Chaturvedi et al., 2009), the Mediator complex subunits Med1, Med12 and Med17 were enriched on the promoter of the active *β* ^*major*^ *gene* in differentiating erythroid cells and a significant reduction in the binding of these factors was observed upon G9a knockdown (Figures 5B–D). Interestingly, a similar decrease in the binding of Mediator complex subunits Med1, Med12, and Med17 on the active *β* ^*major*^ *gene* promoter was observed upon NSD3 knockdown even though G9a was physically present on this gene (Figures 5B–D). These results demonstrate that NSD3 is essential for stabilizing the RNA Pol II and mediator complex binding on the promoter region of G9a target active *β* ^*major*^ *gene*.

While the ChIP results demonstrate that NSD3 is vital for G9a coactivator function on the *β* ^*major*^ *globin* gene, we aim to validate whether NSD3 associates G9a with the coactivator Mediator complex and RNA Pol II *in vivo.* We identified that the immunoprecipitation pulldown of G9a in NSD3 knockdown cells disrupts the association of the coactivator Mediator complex and RNA Pol II with G9a, highlighting that NSD3 is necessary for their *in vivo* association with G9a (Supplementary Figure S5A). The observation is further strengthened by the fact that the association of NSD3 with the Mediator complex and RNA Pol II is intact even when G9a is depleted (G9a KD condition) in the cells (Supplementary Figure S5B). These results collectively show that NSD3 is vital for forming the G9a coactivator complex, and it associates the G9a-GLP complex with the pre-initiation complex (PIC) components, RNA Pol II, and the Mediator complex *in vivo*.

### NSD3 stabilizes the localization of H3K36 trimethylation and RNA polymerase on the active G9a target *β* ^*major*^ *globin* gene

**FIGURE 6:**
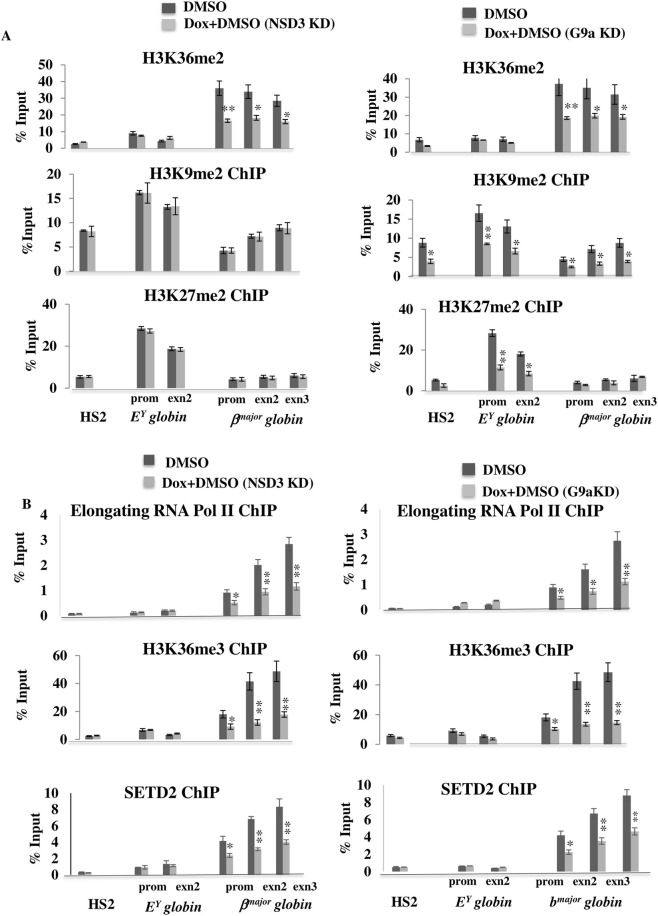
NSD3, associated with SETD2 to mediate activation of the G9a target *β* ^*major*^ *globin* gene via H3K36 di/tri-methylation. **(A)** ChIPs were performed after differentiation in G9a/NSD3-depleted (Diff.) vs. normal (Diff.) MEL cells to analyze the enrichment of H3K36Me2, H3K9Me2, and H3K27Me2 on LCR HS2, repressed *E* ^*Y,*^ and active *β* ^*major*^ *globin* genes. ChIPs were detected by real-time qPCR using specific primers and probes located at the HS2 site of the locus control region, as well as the promoter and gene body of *E* ^*Y*^ *globin* and *β* ^*major*^ *globin genes*. **(B)** Native ChIPs and crosslinked ChIP were performed to measure the levels of enrichment of H3K36me3 and elongating RNA Pol II, SETD2, after differentiation in NSD3 or G9a-depleted (Diff. NSD3/G9a KD) vs. normal (Diff.) MEL cells. ChIPs were detected by real-time qPCR using specific primers and probes located at the HS2 site of the locus control region, as well as the promoter and gene body of *E* ^*Y*^ *globin* and *β* ^*major*^ *globin* genes. Error bars represent SDs calculated from triplicate experiments. *P < 0.05; **P < 0.01.

NSD3 is localized to the *β* ^*major*^ *globin* gene in a G9a-dependent manner, and the knockdown of NSD3 or G9a significantly reduces the binding of NSD3 to this gene. Hence, we next asked whether NSD3-mediated H3K36 dimethylation is required to activate the G9a target *β* ^*major*^ *globin* gene. We performed native H3K36me2 ChIP in NSD3-and G9a-knockdown differentiated erythroid cells. ChIP results showed a significant increase in H3K36me2 marks on the active *β* ^*major*^ *globin* gene promoter and coding region in differentiated erythroid cells. Depletion of NSD3 results in decreased H3K36me2 on the *β* ^*major*^ *globin* gene, highlighting the necessity of these marks in maintaining *β* ^*major*^ *globin* gene activation (Figure 6A). Since NSD3 binding on *β* ^*major*^ *globin* gene is dependent on G9a, a decrease in the localization of the H3K36me2 mark was also observed upon G9a knockdown (Figure 6A). Furthermore, depletion of G9a leads to a reduced level of H3K9me2 on HS2, *E* ^*Y*^ *globin,* and *β* ^*maj*^ *globin* gene, and H3K27me2 marks on the *E* ^*Y*^ *globin* gene as previously reported (Chaturvedi et al., 2009) whereas NSD3 knockdown does not affect levels of H3K9me2 and H3K27me3 (Figure 6A). These results suggest NSD3 mediate deposition of H3K36me2 mark on G9a target adult *β* ^*major*^ *globin* gene in differentiated erythroid cells.

G9a promotes transcription elongation by associating with elongating RNA Pol II (Yuan et al., 2007) and stabilizing localization of H3K36me3 on the coding region of the activated adult *β* ^*major*^ *globin* (Chaturvedi et al., 2009). Similarly, NSD3 also promotes transcription elongation through its association with elongating RNA Pol II (Jeong et al., 2020); hence, we tested whether NSD3 depletion affects H3K36me3 and the localization of elongating RNA Pol II on this gene, as observed with G9a. We found NSD3 knockdown led to a significant decrease in the localization of both H3K36me3 marks and elongating RNA Pol II on the *β* ^*major*^ globin gene, like G9a (Figure 6B). Further, we also found that the localization of H3K36me3 was accompanied by binding of H3K36me3 methyltransferase SETD2 and knockdown down of either G9a or NSD3 lead to decrease in SETD2 and H3K36 trimethylation (Figure 6B), suggesting that the G9a-NSD3 complex regulate the localization of H3K36me3 on the active *β* ^*major*^ *globin* gene by facilitating the binding of H3K36 trimethyl transferase SETD2 on this gene.

NSD3 is localized to the β^major globin gene in a G9a-dependent manner, and knockdown of either NSD3 or G9a significantly reduces NSD3 binding at this locus. We next asked whether NSD3-mediated H3K36 dimethylation is required for activation of the G9a target β^major globin gene. To address this, we performed native H3K36me2 ChIP in differentiated erythroid cells following NSD3 or G9a knockdown. ChIP analysis revealed a significant enrichment of H3K36me2 at the promoter and coding region of the active β^major globin gene in differentiated erythroid cells. Depletion of NSD3 resulted in a marked reduction of H3K36me2 at the β^major globin gene, highlighting the importance of this modification in maintaining gene activation (Figure 6A). Because NSD3 binding at the β^major globin gene is dependent on G9a, a corresponding decrease in H3K36me2 localization was also observed upon G9a knockdown (Figure 6A). Furthermore, depletion of G9a led to reduced H3K9me2 levels at HS2, Ey globin, and β^major globin genes, as well as reduced H3K27me2 at the Ey globin gene, as previously reported (Chaturvedi et al., 2009), whereas NSD3 knockdown did not affect H3K9me2 or H3K27me3 levels (Figure 6A). Together, these results suggest that NSD3 mediates deposition of H3K36me2 on the G9a target adult β^major globin gene in differentiated erythroid cells.

G9a has been reported to associate with transcriptionally engaged RNA Pol II and to stabilize H3K36me3 localization across the coding region of the activated adult β^major globin gene (Chaturvedi et al., 2009; Yuan et al., 2007). Similarly, NSD3 has been implicated in transcriptional progression through its association with RNA Pol II (Jeong et al., 2020). We therefore examined whether NSD3 depletion affects H3K36me3 levels and RNA Pol II occupancy across the β^major globin gene, as observed upon G9a loss. We found that NSD3 knockdown resulted in a significant reduction in both H3K36me3 enrichment and RNA Pol II occupancy across the β^major globin gene, comparable to the effects observed following G9a depletion (Figure 6B). In addition, H3K36me3 enrichment was accompanied by binding of the H3K36 trimethyltransferase SETD2, and knockdown of either G9a or NSD3 led to reduced SETD2 occupancy and H3K36 trimethylation (Figure 6B). These findings suggest that the G9a–NSD3 complex regulates H3K36me3 localization on the active β^major globin gene by facilitating recruitment of the H3K36 trimethyl transferase SETD2.

### SETD2 is part of the G9a-NSD3 coactivator complex and mediates H3K36 trimethylation on the active *β* ^*major*^ *globin gene*

**FIGURE 7:**
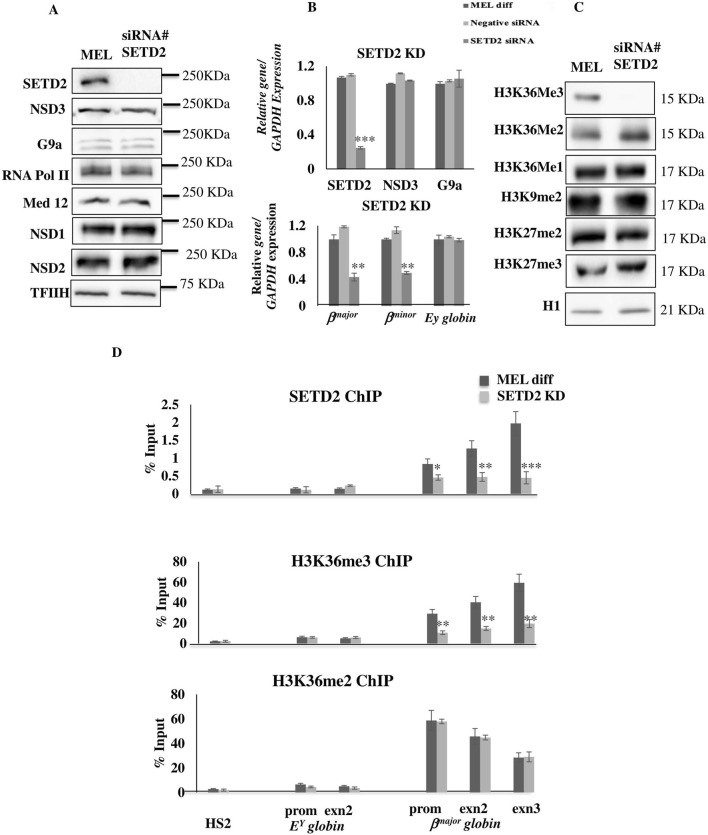
SETD2 knockdown leads to decreased localization of SETD2-mediated H3K36Me3 on adult *β* ^*major*^ *globin* gene. **(A)** The levels of SETD2 in control vs. siRNA SETD2 cells were analyzed at the protein level by Western blot of nuclear extracts using the Abs indicated (Left) and molecular masses (in kilodaltons) are indicated (Right). MEL parent represents a control cell line with no SETD2 siRNA induction. **(B)** The levels of SETD2 in MEL Control, negative siRNA, and SETD2 siRNA were analyzed at the transcript level by RT-qPCR. Transcript levels are normalized to GAPDH with the ratio observed in the absence of Dox set to 1. **(C)** The levels of histone marks in control vs. siRNA SETD2 cells were analyzed at the protein level by Western blot of nuclear extract using the indicated Abs (Left). MEL parent represents a control cell line with no induction of SETD2 siRNA. Molecular masses of proteins (in kilodaltons) are indicated on the right. (Right). **(D)** Crosslinked and Native ChIPs were performed to measure the enrichment levels of SETD2, H3K36Me3, and H3K36Me2 after differentiation in SETD2 siRNA-treated cells versus normal (Diff.) cells. MEL cells. ChIPs were detected by real-time qPCR using specific primers and probes located at the HS2 site of the locus control region, as well as the promoter and gene body of *E* ^*Y*^ *globin* and *β* ^*major*^ *globin* genes. Error bars represent SDs calculated from triplicate experiments. *P < 0.05; **P < 0.01, ***P < 0.001.

While knockdown of G9a and NSD3 reduces the localization of SETD2 and H3K36me3 at the active *β* ^*major*^ *globin* gene, we investigated whether SETD2, the only H3K36 trimethyl transferase, is part of the G9a-NSD3 coactivator complex in differentiated erythroid cells. To validate this, we first tested for SETD2 in G9a, GLP, NSD3, and Mediator IP pulldown assays. Western blotting confirmed that SETD2 is part of the G9a coactivator complex (Figures 1A,D), which was also evident from gel filtration chromatography, where SETD2 co-migrated with G9a-GLP coactivator complex members, including NSD3, Mediator complex proteins, and RNA Pol II (Figure 1C). Further, we also found that the association of G9a, NSD3, and SETD2 is conserved from human to mouse (Supplementary Figure S1). Next, to study whether SETD2 is directly involved in regulating the expression of the G9a-NSD3 target adult *β-globin* genes. We performed siRNA-mediated knockdown of this protein in differentiated erythroid cells. The siRNA-mediated knockdown resulted in more than 90% knockdown of SETD2 at both the transcript and protein levels without altering the expression of G9a, GLP, or NSD histone methyltransferases (Figures 7A,B).

Further, while knockdown of SETD2 did not affect the proliferation of MEL cells, a significant decrease in hemoglobinization was observed in differentiated MEL cells, consistent with the phenotype observed upon G9a/NSD3 knockdowns (Supplementary Figure S6). This decrease in hemoglobinization was accompanied by significant downregulation of the G9a-NSD3 target adult *β* ^*major*^ and *β* ^*minor*^ *globin* genes, suggesting that SETD2 is vital for maximal activation of these genes (Figure 7B). We also observed that SETD2 KD did not affect the expression of most of the genes, including the genes involved in heme biosynthesis *(FECH, PBGD, ALAS1* and *ALAS2)* and erythroid differentiation (*GATA1, EKLF, FOG1,* and *NF-E2) genes* (Supplementary Figure S3), suggesting that similar to G9a and NSD3, SETD2 is exclusively involved in regulation erythroid differentiation by regulating adult *β* ^*major*^ *and β* ^*minor*^ *globin* genes in differentiated erythroid cells. Furthermore, the knockdown of SETD2 significantly reduced the global level of H3K36me3 in cells without altering other histone marks, including H3K36me2, thus highlighting that SETD2 specifically mediates H3K36me3 in differentiated MEL cells (Figure 7C). ChIP assay using SETD2 abs revealed the localization of SETD2 on the active *β* ^*major*^ *globin* gene predominantly on the coding region, and knockdown of SETD2 leads to a significant decrease in its localization on *this* gene. Furthermore, the decrease in the expression of SETD2 upon knockdown resulted in the depletion of the H3K36me3 mark on *β* ^*major*^ *globin,* without affecting NSD3 mediated H3K36me2 marks (Figure 7D). Since H3K36 methylation works antagonistically to the repressive H3K27me3 methylation, we subsequently investigated whether the reduction in H3K36me2/me3 following G9a or NSD3 knockdowns is concomitant with an elevation in repressive H3K27me3 marks on the *β* ^*major*^ *globin* gene. The ChIP experiment revealed that a decrease in H3K36me2/me3 marks was accompanied by a significant increase in the presence of the repressive H3K27me3 mark on the *β* ^*major*^ *globin* gene, specifically at the promoter region (Supplementary Figure S7). Overall, these results suggest that SETD2 is part of the G9a-NSD3 coactivator complex, and that a coordinated mechanism among the three histone methyltransferases is necessary to activate adult *β-globin* genes in differentiated erythroid cells.

## Discussion

### NSD3-G9a crosstalk is vital for G9a coactivator complex formation

The mechanisms of G9a-mediated gene repression are well documented; however, a few studies have focused solely on G9a′s positive regulation of gene expression (Poulard et al., 2021). The coactivator function of G9a is independent of its enzymatic activity and depends on its interactions with other proteins. For example, G9a associates with GRIP1, CARM1, and p300 to function as a coactivator for glucocorticoid receptor (GR)- regulated genes (Bittencourt et al., 2012). Alternatively, G9a forms coactivator complexes with histone acetyltransferases to form coactivator complexes like G9a/PHF20/MOF and G9a/E2F1/PCAF complexes required to activate ERa and E2F1 regulated genes, respectively (Rao et al., 2016; Zhang et al., 2016). In differentiated erythroid cells, the G9a forms a coactivator complex with a Mediator complex. It mediates the activation of adult *β* ^*-*^ *globin* genes by stabilizing pre-initiation complex formation and H3K36 methylation on the promoter and coding regions, respectively (Chaturvedi et al., 2012). However, the exact molecular mechanism by which G9a stabilizes Mediator complex binding and regulates H3K36 methylation remained unclear. Here, we demonstrate that G9a coordinates with H3K36 histone methyltransferases NSD3 and SETD2 to mediate these functions in an NSD3-dependent manner. G9a directly interacts with NSD3, wherein NSD3 plays a vital role in forming the G9a coactivator complex. This assembly involves the association of the G9a/GLP complex with the Mediator complex, RNA Pol II, and SETD2 histone methyltransferase. Knockdown of NSD3 in differentiated erythroid cells, followed by G9a IP, revealed that NSD3 depletion destabilizes the association of the G9a/GLP complex with the Mediator complex, RNA Pol II, and SETD2, highlighting the necessity of NSD3 in stabilizing the G9a coactivator complex. While NSD3 is shown to be part of the G9a/LSD2/AOF1/KDM1b complex that facilitates transcription elongation on active genes (Fang et al., 2010), and HAT/RNA Pol II coactivator complex, which facilitates transcription activation (Jeong et al., 2020), however, the G9a/GLP coactivator complex comprising of H3K36 methyltransferases NSD3 and SETD2, along with the Mediator complex reported in the current study, has not been described previously. Furthermore, an interaction between the three methyltransferases from murine to human cells suggests that the G9a coactivator complex is conserved from humans to mice and possibly plays an essential role in activating a specific set of genes, potentially those involved in key biological processes such as development or cell differentiation. Nonetheless, additional studies are warranted to precisely identify which target genes are regulated by this complex in human and mouse cells and to clarify its biological significance further.

### NSD3-G9a crosstalk is vital for gene activation and mediator complex binding on G9a target genes

NSD3 belongs to the NSD family of H3K36 histone methyltransferases, catalyzing histone H3K36 dimethylation and mediating gene activation by promoting transcription initiation and elongation of active genes in association with RNA Pol II (Jeong et al., 2020; Fang et al., 2010). G9a activates transcription of target *β* ^*major*^ *globin* and *β* ^*minor*^ *globin* genes by facilitating pre-initiation complex (PIC) formation through stabilization of RNA Pol II and mediator complex binding on promoter (Shankar et al., 2013). The current study shows that G9a mediates these functions through NSD3. G9a associates with NSD3 and recruits it to the promoter of G9a target genes, including adult *β-globin* genes. NSD3 mediates the activation of these genes by stabilizing RNA Pol II and promoting the binding of the mediator complex to the promoter. NSD3 knockdown in differentiated erythroid cells destabilizes the mediator complex and RNA Pol II binding at the promoter region, resulting in significant downregulation of the G9a target *β* ^*major*^ *globin* and *β* ^*minor*^ *globin* genes. This destabilization of the mediator complex upon NSD3 or G9a knockdown was also accompanied by a decrease in H3K36 dimethylation, predominantly in the promoter region of the *β* ^*major*^ *globin* gene, suggesting that NSD3-mediated H3K36me2 is vital for the activation of the G9a target *β* ^*major*^ *globin* genes. These findings corroborate previous studies reporting that NSD3-mediated H3K36 dimethylation predominantly localizes to the promoter regions of active genes (Jeong et al., 2020; Shipman et al., 2024).

Since NSD3 depletion did not affect G9a localization at the *β* ^*major*^ *globin gene*, a significant decrease in its expression was observed, suggesting that NSD3 is vital for stabilizing the coactivator Mediator complex and RNA Pol II binding at the promoter regions of G9a target genes. This observation was further supported by immunoprecipitation pulldown, which revealed that NSD3 mediates the association of the Mediator complex and RNA Pol II with the G9a/GLP complex, and that NSD3 depletion destabilizes the interaction between the G9a and Mediator-RNA Pol II complex. We speculate that NSD3 functions as a bridge, facilitating protein-protein interactions that associate G9a with the coactivator Mediator complex and the basal transcription machinery, including RNA Pol II, thereby mediating G9a coactivator function and activating the adult *β-globin* genes. A similar mechanism for glucocorticoid receptor-regulated genes has been reported, in which G9a recruits the coactivators CARM1 and p300 to GR target genes to mediate gene activation (Bittencourt et al., 2012). However, further investigation is required to elucidate the mechanisms regulating the association of the G9a-NSD3-Mediator complex. Distinct mechanisms involving post-translational modifications, such as the auto-methylation of G9a (Poulard et al., 2017), the sumoylation of the N-terminal region of G9a (Srinivasan et al., 2019), or the methylation of interacting partners of the coactivator complex by G9a/GLP to facilitate recruitment to target genes (Zhang et al., 2016), have been reported. It will be interesting to explore whether the formation and activation of the G9a-NSD3 coactivator complex involve similar mechanisms.

### G9a-NSD3 crosstalks with H3K36 trimethyl transferase SETD2 to mediate activation of G9a target genes

G9a localizes to transcriptionally active genes, with increased localization at the 3′end of the coding region. It has been speculated that functionally, G9a-mediated H3K9me2 suppresses cryptic transcript formation and facilitates transcription elongation of these genes (Vakoc et al., 2005). Similarly, H3K36me3 has been shown to promote transcriptional elongation of active genes in association with RNA Pol II (Rahman et al., 2011). In agreement with these studies, we also observed that both marks were predominantly localized in the coding region *o*f the *β* ^*major*^ globin gene, with greater enrichment towards the 3′end. SETD2 is an H3K36 methyltransferase primarily localized in the coding regions of active genes, with the peak accumulation at the 3′end. It facilitates the activation of these genes through association with elongating RNA Pol II (Bhattacharya and Workman, 2020). It is responsible for trimethylating H3K36 (H3K36me3), a histone modification associated with actively transcribed genes (Molenaar and van Leeuwen, 2022). SETD2’s role in establishing H3K36me3 is critical for maintaining proper transcriptional elongation and preventing spurious transcriptional initiation from cryptic promoters (Bhattacharya and Workman, 2020). We have previously reported that G9a stabilizes the localization of H3K36me3 on the G9a target *β* ^*maj*or^ globin gene, and depletion of G9a significantly affects the localization of H3K36me3 marks on the coding region of G9a target adult *β-globin* genes (Chaturvedi et al., 2009), speculating the possible association of H3K36 trimethyl transferase with G9a coactivator complex. The present study refines this mechanism, demonstrating that G9a facilitates the localization of H3K36me3 by associating with the H3K36 trimethyl transferase SETD2. Our data suggest that SETD2 is part of the G9a-NSD3-Mediator coactivator complex, and NSD3 is crucial in associating SETD2 with the G9a coactivator complex. Depletion of NSD3 destabilizes the association of SETD2 with the G9a coactivator complex, leading to decreased localization of H3K36me3 and elongating RNA Pol II in the coding region of the G9a target *β* ^*major*^ globin gene, even though G9a is physically present on this gene. Further knockdown of SETD2 in differentiated erythroid cells results in a global reduction of H3K36me3 and a decrease in the localization of this mark on the G9a target *β* ^*major*^ globin, accompanied by decreased expression of adult *β-*globin genes. These results suggest that SETD2 is necessary for maximal activation of the G9a target adult *β-*globin genes.

It is well documented that H3K36 methylation acts in opposition to H3K27 trimethylation (Lam et al., 2022). Corroborating these results, we find that the interplay between NSD3 and SETD2 antagonizes the repressive H3K27 trimethylation marks on the adult *β−globin* genes and ensures robust, sustained transcriptional activation required for their expression in differentiated erythroid cells. Overall, these results reveal new mechanistic insights into the role of G9a-mediated gene activation, where G9a associates with NSD3 and SETD2 to mediate H3K36 di and trimethylation at the regulatory region and gene body, respectively, and facilitates transcription initiation and elongation required for the maximal expression of G9a target adult *β-globin* genes.

## Conclusion

**FIGURE 8:**
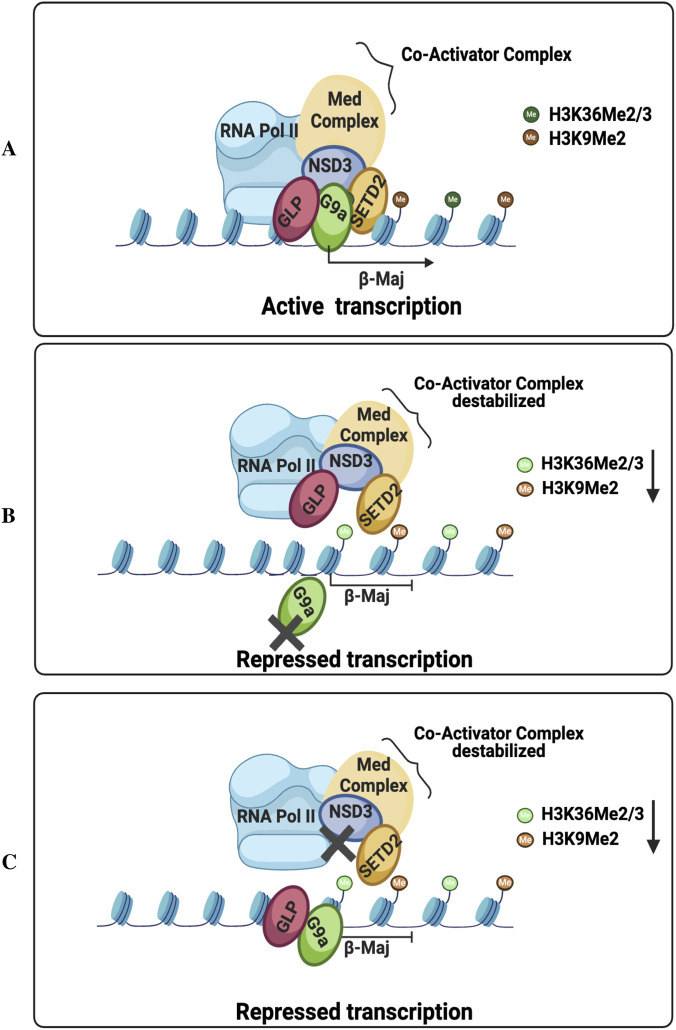
Model for G9a mediated coactivator function. **(A)** G9a physically interacts with NSD3, recruits it on G9a target *β* ^*major*^ *globin* gene, and NSD3 mediates the activation of this gene by facilitating mediator complex binding, H3K36me2 on the promoter region and by facilitating SETD2 mediated H3K36 me3 in the coding region. **(B)** Depletion of G9a destabilizes NSD3 binding resulting in decreased localization of NSD3 accompanied by decreased Mediator complex, NSD3 mediated H3K36me2 and SETD2 mediated H3K36me3 binding on G9a target *β* ^*major*^ *globin* gene, suggesting G9a is necessary for recruitment/stabilization of NSD3 on G9a target gene. **(C)** Depletion of NSD3 downregulates the localization of the mediator complex binding, as well as the NSD3-and SETD2-mediated H3K36me2 and H3K36me3 marks on the G9a target *β* ^*major*^ *globin* gene, even though G9a is physically present on this gene, suggesting that NSD3 is vital for the formation of the G9a coactivator complex and for G9a-mediated gene activation.

Our study demonstrates a coordinated role for G9a, NSD3, and SETD2 in gene regulation, introducing a new model for G9a-mediated coactivator function (Figure 8, model). We show that NSD3 and SETD2 are essential partners of the G9a-Mediator coactivator complex and are necessary for maximal expression of G9a target adult *β-globin* genes. NSD3 promotes the activation of G9a target genes by facilitating transcription initiation and elongation. It stabilizes RNA Pol II and the Mediator complex at the promoter and facilitates SETD2-mediated H3K36 trimethylation in the gene body. Since the G9a-NSD3-SETD2 interaction is conserved from mouse to human cells, we speculate that the crosstalk of the G9a/GLP complex with NSD3 and SETD2 is vital for the activation of a set of genes in these cells. Though further studies focusing on the functional co-localization of G9a, NSD3, and SETD2 at the genome-wide level will be vital to get deeper insight into the regulatory mechanism by which cross-talk with NSD3 and SETD2 histone methyltransferases and mediates gene activation.

## Data Availability

The original contributions presented in the study are included in the article/Supplementary Material, further inquiries can be directed to the corresponding author.
