# Supplementary material for: Histone methyltransferase G9a crosstalks with H3K36 histone methyltransferases NSD3 and SETD2 to mediate gene activation
Source: Front Cell Dev Biol. 2026 Mar 26;14:1790894. doi: 10.3389/fcell.2026.1790894 (PMC13062286; doi:10.3389/fcell.2026.1790894)
Supplement: Supplementary file 1 [file DataSheet1.pdf]

Figure-S1

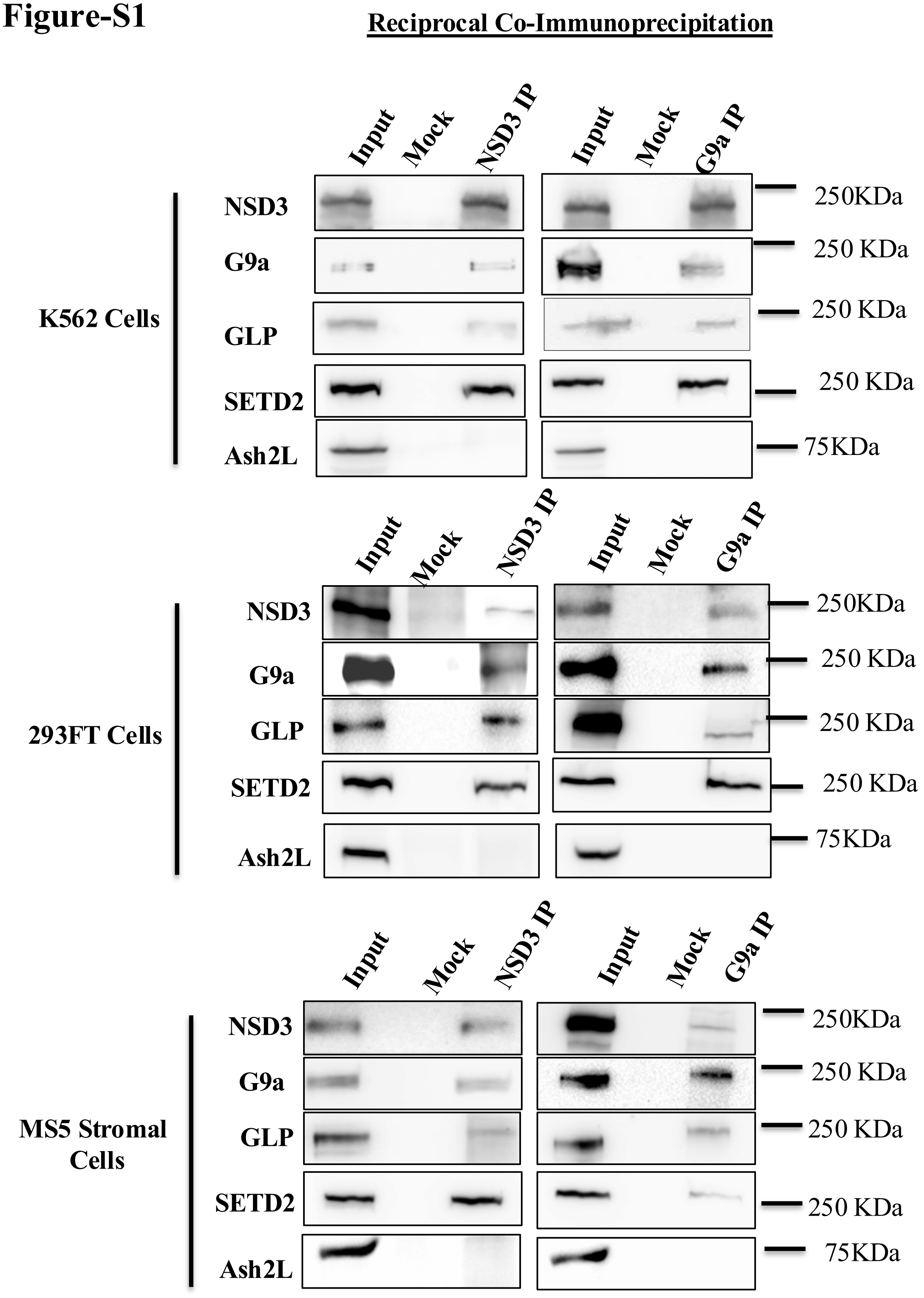

Fig. S1: The interaction of NSD3/SETD2 with the G9a/GLP complex is conserved in humans to mice. Proteins immunoprecipitated via Abs against NSD3 and G9a were analyzed by Western blot in human K562 cells, human 293FT cells, and murine MS-5 stromal cells. A mock IP with normal IgG was used as a negative control. Abs used for Western blot (Left) and molecular masses (Right; in kilodaltons) are indicated.

Figure-S2

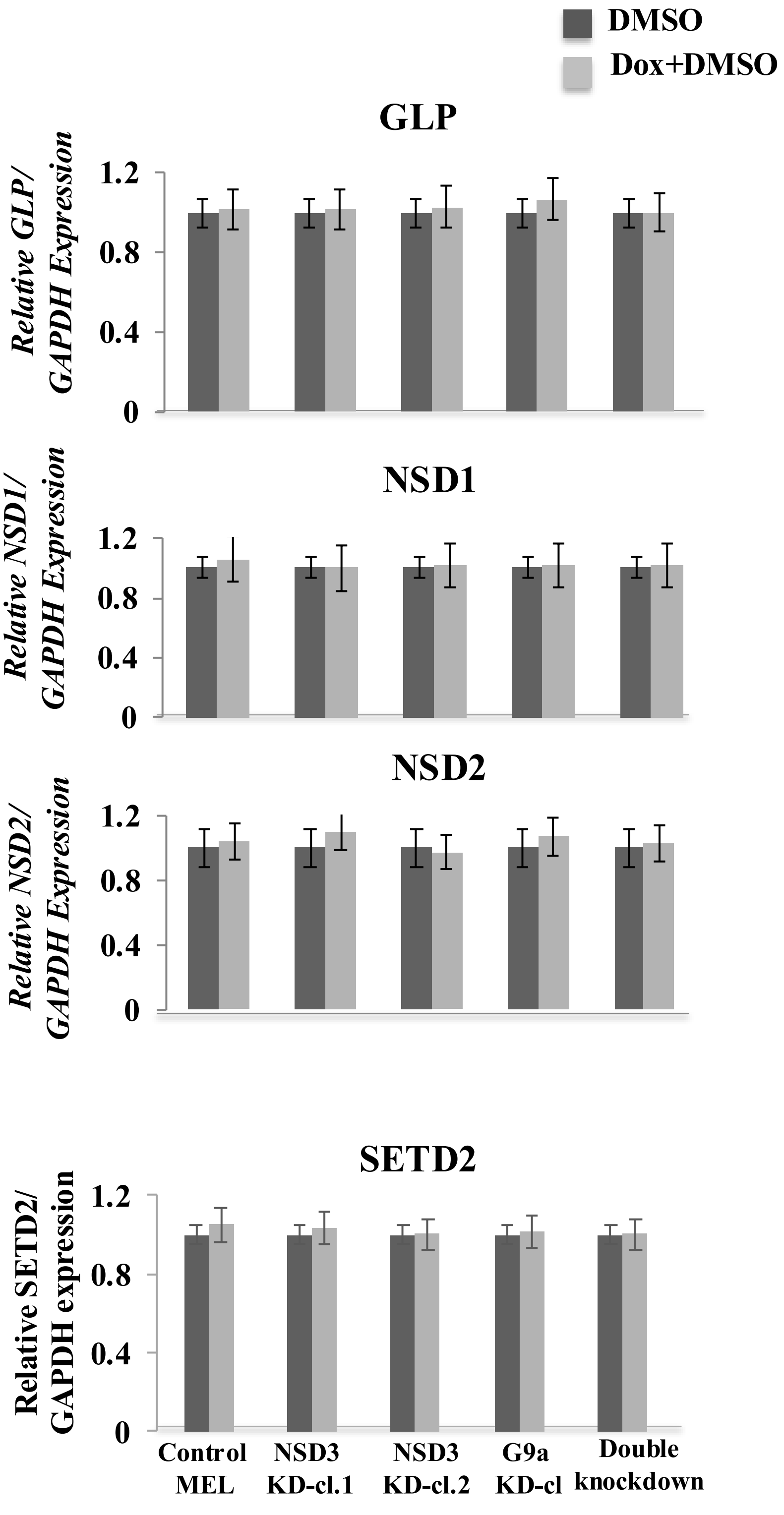

Fig. S2: NSD3 or G9a or G9a plus NSD3 downregulation does not affect the expression of GLP, NSD1, NSD2 and SETD2 histone methyltransferases. Transcription of GLP, NSD1, NSD2 and SETD2 genes were assessed by RT-qPCR after differentiation in NSD3 or/and G9a-depleted (Dox) vs. normal (No Dox) MEL cells. Transcripts are expressed relative to GAPDH with the highest ratio set to 1. Average values ± SD represent three independent experiments. Non- significant difference was observed in DMSO vs Dox DMSO conditions

Figure-S3

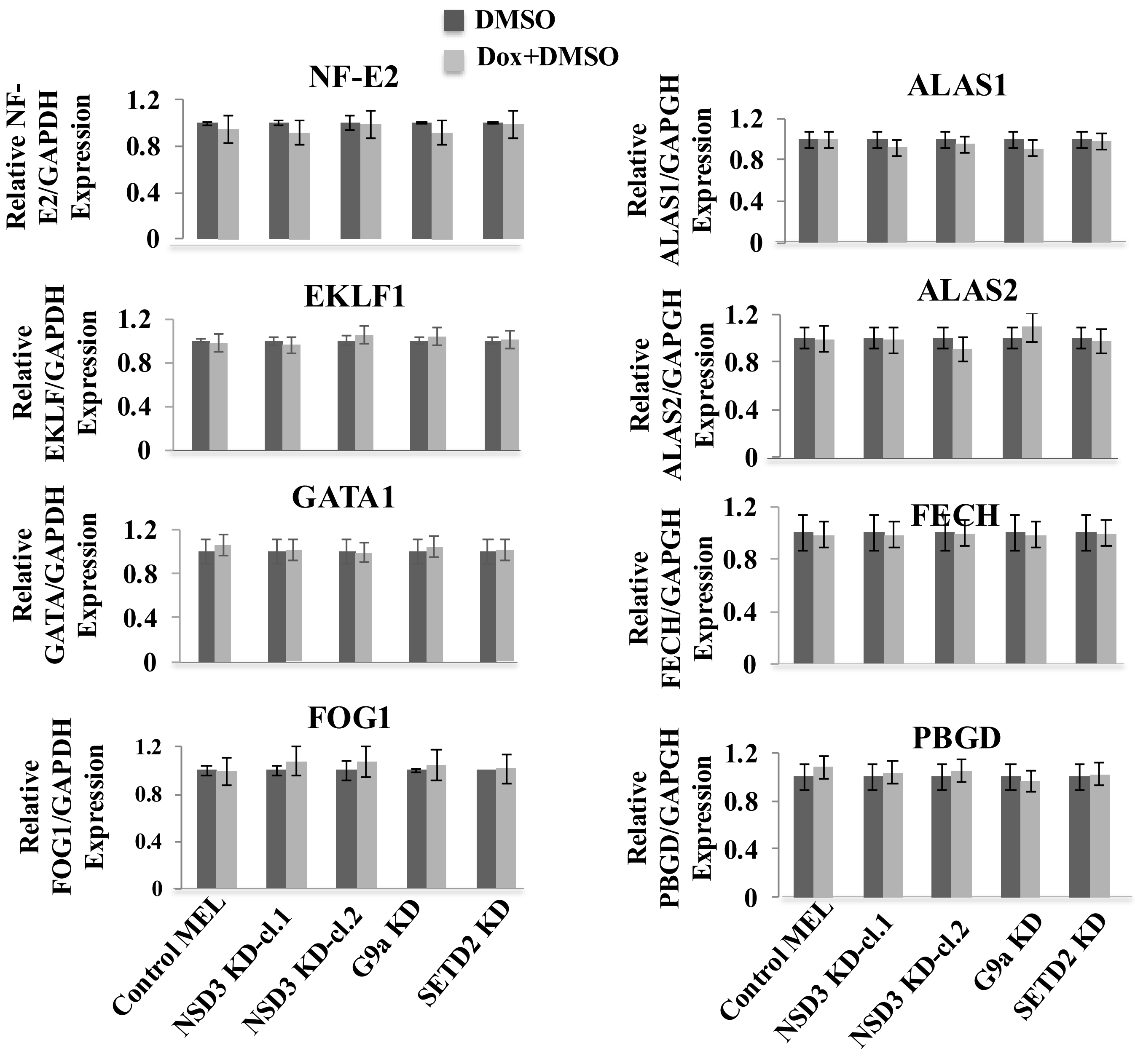

Figure S3: NSD3, G9a, or SETD2 knockdown does not alter the expression of erythroid-specific transcription factors and heme biosynthesis associated genes. KD of NSD3 or G9a does not alter the expression of erythroid-specific transcription factors (viz. *GATA1, EKLF, FOG1,* and *NF-E2)* or heme biosynthetic pathway associated genes (viz. *FECH, PBGD, ALAS1,* and *ALAS2)* in differentiated erythroid cells. RT-qPCR was performed to analyze the effect of NSD3 KD on other genes. Transcripts values are expressed relative to GAPDH, with the highest ratio set to 1. Average values from triplicate experiments are represented with error bars corresponding to SDs. Non-significant difference was observed in DMSO vs Dox DMSO conditions

Figure S4

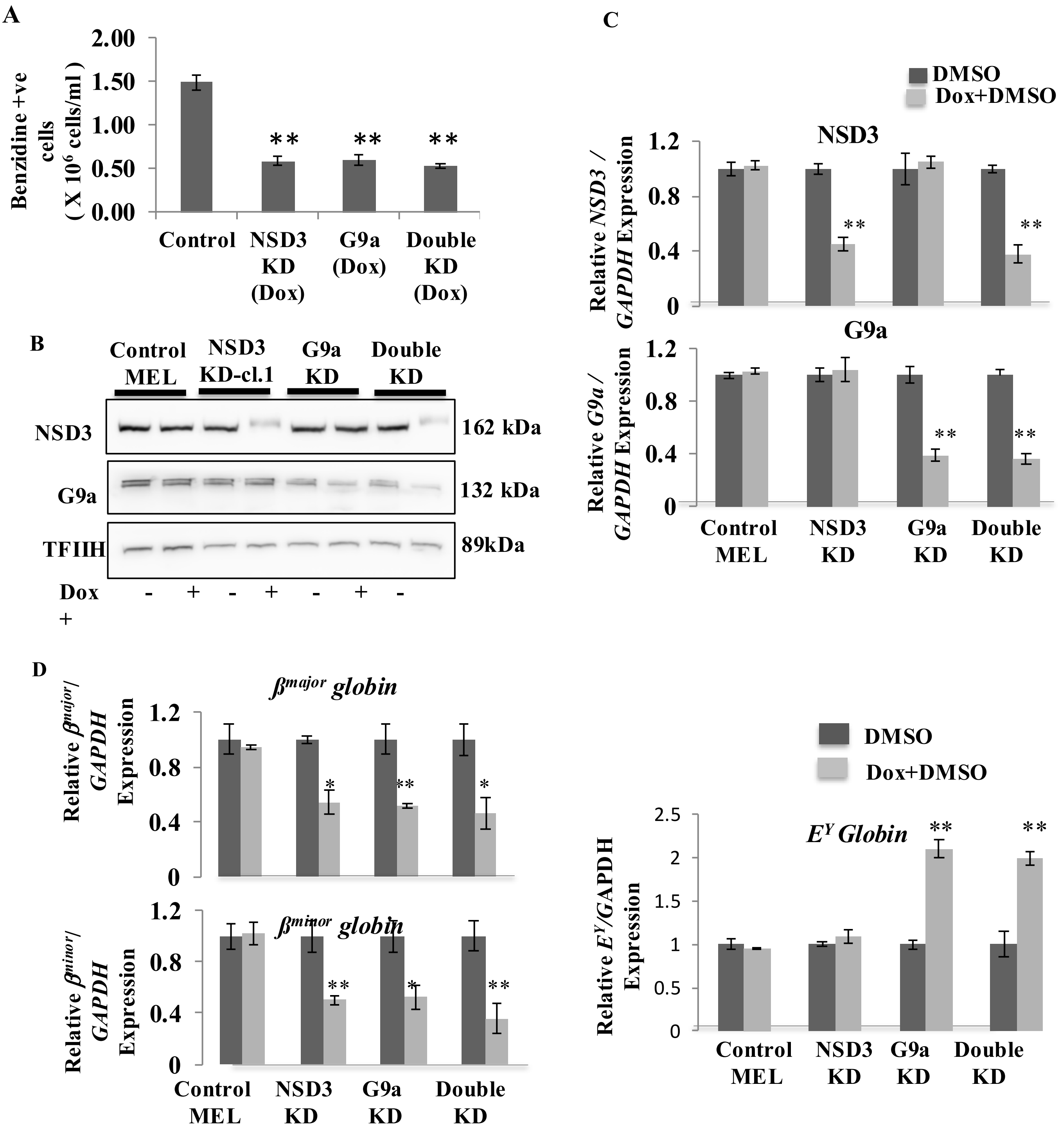

Fig. S4: Development of a Dox inducible G9a plus NSD3 double knockdown MEL cell line. (*A*) NSD3, G9a KD or G9a-NSD3 double knockdown decreases hemoglobinization in erythroid cells. Quantitative representation of benzidine positive cells in NSD3, G9a, and NSD3 plus G9a depleted cells vs. Control MEL cells in differentiated condition. **(B)** The levels of NSD3 and G9a in Dox-Error bars represent SDs calculated from triplicate experiments. *P < 0.05; **P < 0.01.

treated (Dox) vs. untreated (No Dox) cells were analyzed at the protein level by Western blot of nuclear extracts using the indicated Abs (Left). MEL parent represents a control cell line with no induction of shRNA on Dox treatment. Molecular masses of proteins (in kilodaltons) are indicated on the right. **(C)** The levels of NSD3,G9a and G9a-NSD3 double KD in Dox-treated (Dox) vs. untreated (No Dox) cells were analyzed at the transcript level by RT-qPCR. **(D)** The double knockdown of NSD3 plus G9a showed slightly increased silencing of β^{maj0r} *and* β^{minor} *globin* genes compared to single KD of NSD3 or G9a. Transcripts levels in single and double knockdown conditions were measured by real-time RT-qPCR in differentiated Dox-treated/untreated cells. NSD3 KD has no effect on E^{Y} *globin* gene expression as the double knockdown of NSD3 plus G9a showed the same upregulation of E^{Y} *globin* genes when compared to the single KD of G9a. Transcript levels are normalized to GAPDH, with the ratio observed in the absence of Dox set to 1. Average values from triplicate experiments are represented with error bars corresponding to SDs. $^{*}P < 0.05$; $^{**}P < 0.01$.

Figure S5

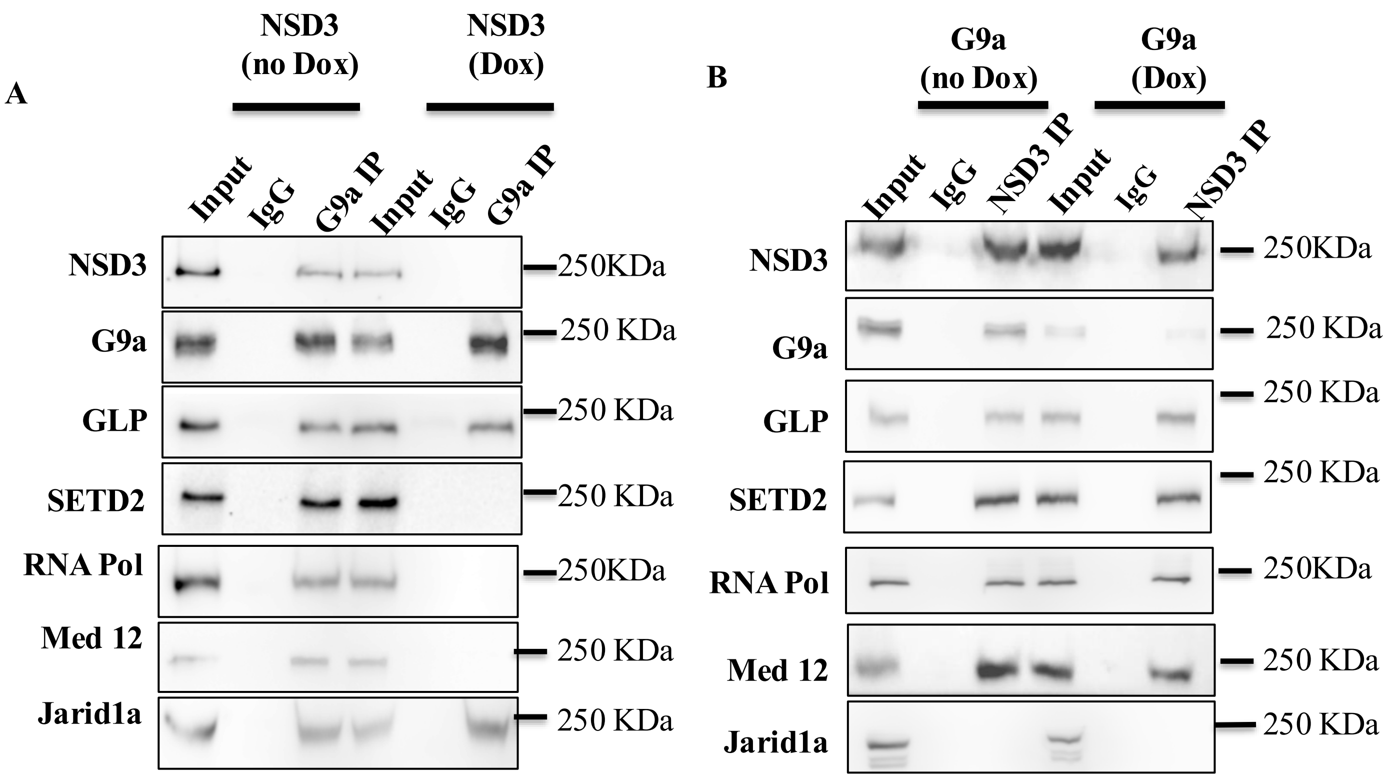

Fig S5: NSD3 is vital for associating the Mediator complex, RNA Pol II and SETD2 with the G9a to form a coactivator complex. Depleting NSD3 disrupts the interaction between G9a and the mediator complex/ RNA Pol II/SETD2. **(A)** G9a and associated proteins were immunoprecipitated from a MEL nuclear extract containing a normal (MEL parent) or reduced amount of NSD3 (NSD3 KD). **(B)** Depletion of G9a (G9a KD) does not disrupt the association of NSD3 with mediator complex, RNA Pol II and SETD2. NSD3 and associated proteins were immunoprecipitated from a MEL nuclear extract containing a normal (MEL parent) or reduced) amount of G9a (G9a KD. Mock IPs with normal IgG were used as negative controls. Immunoprecipitated proteins were analysed by Western blot. Abs used for Western blot (Left) and molecular masses (Right; in kilodaltons) are indicated.

Figure-S 6

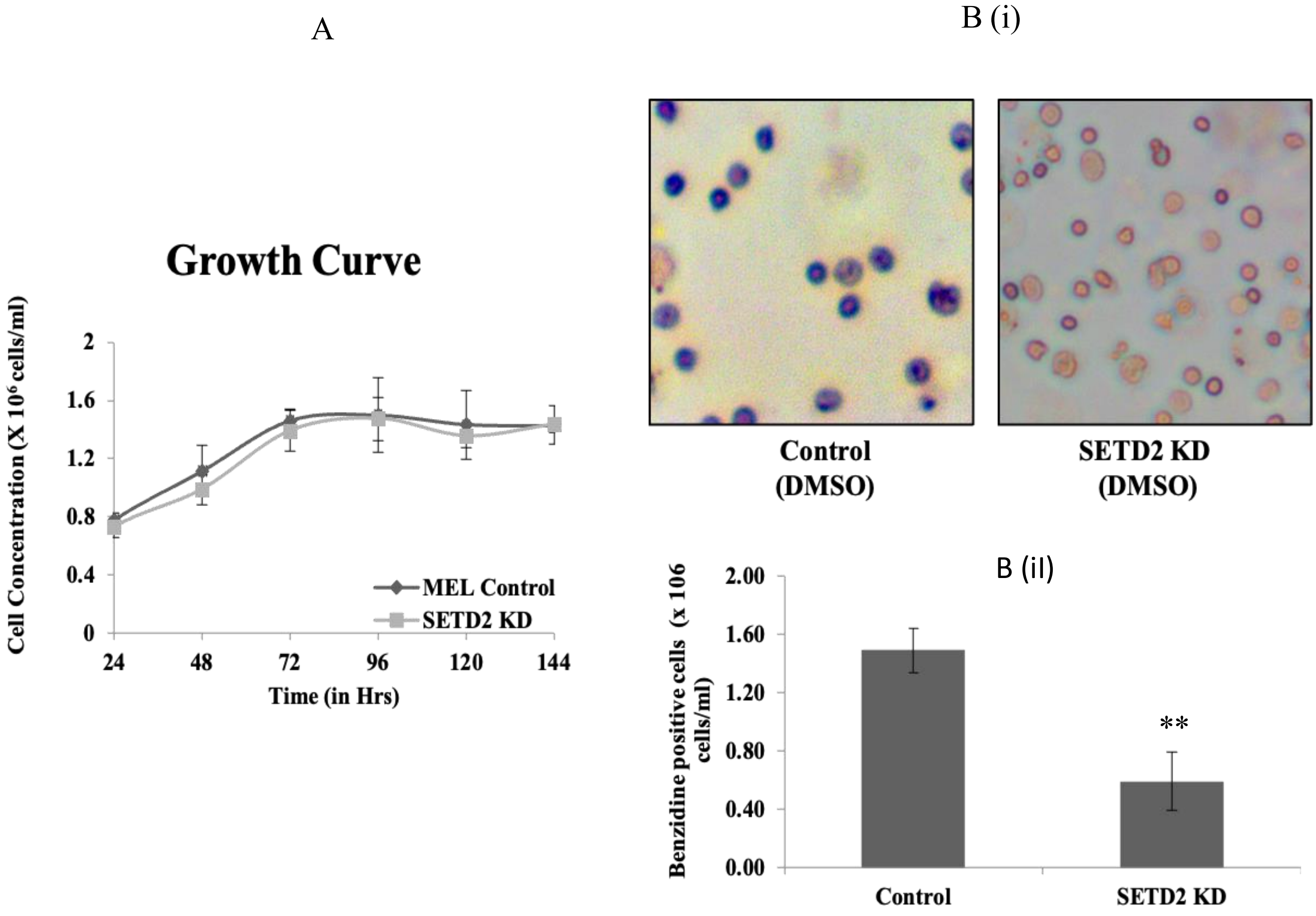

Fig S6: This figure shows the results SETD2 knockdown (KD). **(A)** SETD2 KD does not affect cell growth. Cell concentration was assessed every 24 hours after induction of erythroid differentiation in SETD2 depleted vs. normal MEL cells. ***(B)*** **(i)** SETD2 KD decreases hemoglobinization in erythroid cells. Hemoglobin content was assessed by benzidine staining in NSD3, G9a or SETD2-depleted vs. normal (No Dox) MEL cells after 96 hours of erythroid differentiation. **(B) (ii)**Quantitative representation of benzidine positive cells in SETD2 depleted cells vs. Control MEL cells in differentiated condition. Error bars represent SDs calculated from triplicate experiments. $^{**}P < 0.01$.

Figure-S7

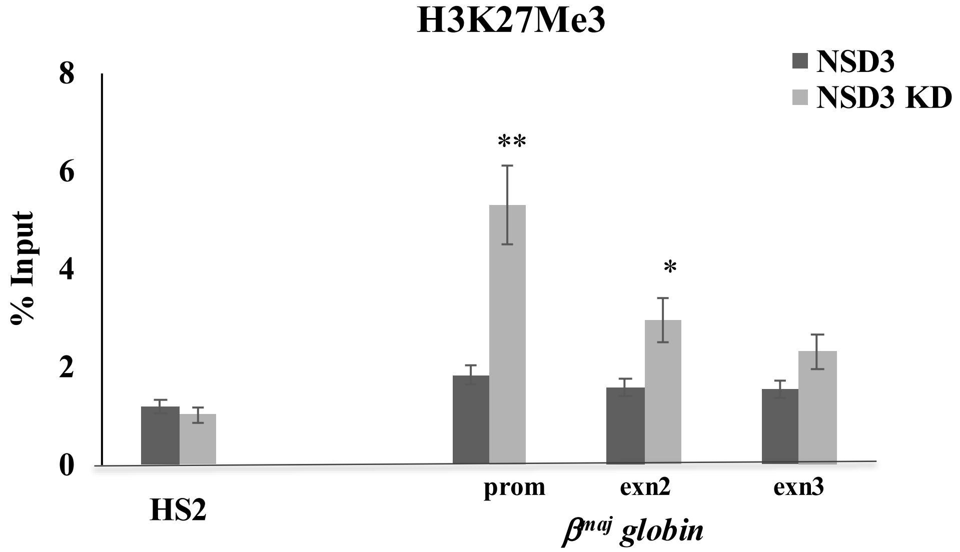

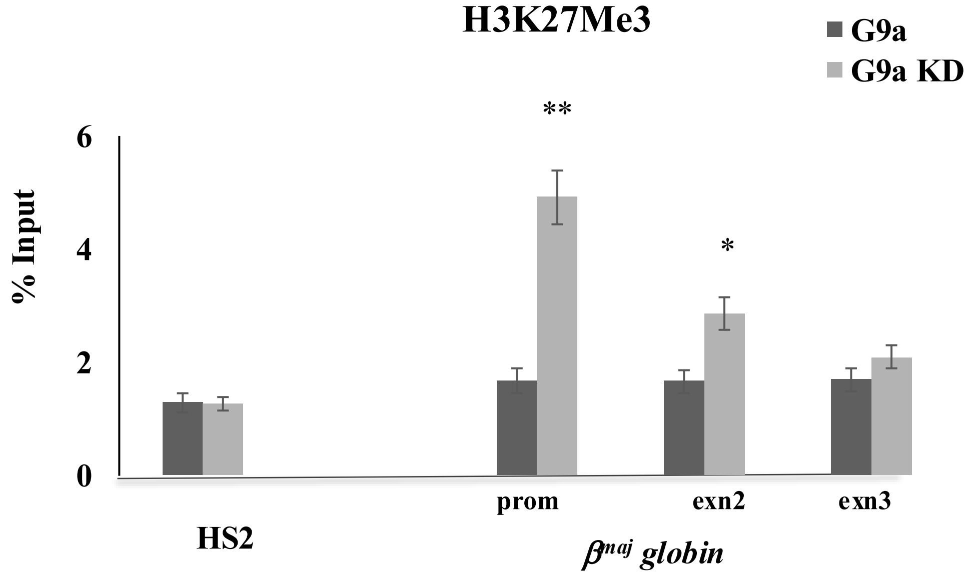

Fig S7: Antagonistic roles of H3K36me2/3 and H3K27me3. Depleting of NSD3 or G9a leads to decreased H3K36Me2/3 (Figure-6 of manuscript), leading to increased repressive H3K27 trimethylation on the β^{major}*-globin* gene thus leading to downregulation of this gene. Native ChIPs were performed to measure the levels of H3K27me3, after differentiation in NSD3 or G9a-depleted (Diff. NSD3/G9a KD) vs. normal (Diff.) MEL cells ChIPs were revealed by real-time qPCR using specific primers and probes located at the HS2 site of the locus control region and promoter and gene body of β^{major} *globin* genes. Error bars represent SDs calculated from triplicate experiments. *P < 0.05; **P < 0.01.
